# Fechner (1866): The Aesthetic Association Principle—A Commented Translation

**DOI:** 10.1177/2041669520920309

**Published:** 2020-05-21

**Authors:** Stefan A. Ortlieb, Werner A. Kügel, Claus-Christian Carbon

**Affiliations:** Department of General Psychology and Methodology, University of Bamberg; Bamberg Graduate School of Affective and Cognitive Sciences; Research Group EPÆG (Ergonomie, Psychologische Æsthetik, Gestalt), Bamberg, Germany; Nürnberg, Germany; Department of General Psychology and Methodology, University of Bamberg; Bamberg Graduate School of Affective and Cognitive Sciences; Research Group EPÆG (Ergonomie, Psychologische Æsthetik, Gestalt), Bamberg, Germany

**Keywords:** empirical aesthetics, aesthetic association principle, Fechner, full text translation

## Abstract

Most of the groundbreaking works of Gustav Theodor Fechner (1801–1887), who paved the way for modern experimental psychology, psychophysics, and empirical aesthetics, are so far only available in German. With the first full text translation of Fechner’s article on *The Aesthetic Association Principle* (*Das Associationsprincip in der Aesthetik*), we want to fill in one of the blank spots in the reception of his Aesthetics from Below (Aesthetik von Unten). In his 1866 article, Fechner devises a fundamental principle that accounts for the role of associations in the formation of aesthetic preferences. Based on concrete everyday examples and thought experiments, he demonstrates how aesthetic choices are largely shaped by the observer’s learning history (associative factors) rather than by an object’s formal properties (direct factors). Fechner’s Aesthetic Association Principle has lost nothing of its initial relevance as the role of content and personal meaning is still grossly underrated in theory and practice of empirical aesthetics today.

## Introductory Notes on Fechner’s Aesthetic Association Principle

By the association principle, I mean a principle, that is already known and recognized in psychology for its significance and its scope, but which is hitherto hardly appreciated in aesthetics. (Fechner, 1876, quoted from Ortlieb & Carbon, 2019, p. 14)

Philosopher and physicist Gustav Theodor Fechner (1801–1887) is famous for pioneering psychophysics and experimental aesthetics, although much of his influential work is still untranslated and therefore inaccessible to many scholars from both disciplines (Scheerer, 1987). On occasion of the Fechner Year 1987, Scheerer showed that this lack of English translations has led to some serious misconceptions and blind spots in the reception of Fechner’s psychophysics. While the *Elements of Psychophysics* (Fechner, 1860/1966) is at least partly available in English, *none* of Fechner’s equally groundbreaking works on aesthetics have so far been translated into English.^1^ This is perhaps one of the reasons why his Aesthetics from Below (Aesthetik von Unten) is still widely mistaken for an application of psychophysics, rather than a full-fledged research programme in its own right. It, of course, entails the application of certain psychophysical elements like the threshold concept (Aesthetische Schwelle), the method of choice (Methode der Wahl), the method of production (Methode der Herstellung), and the method of use (Methode der Verwendung) to aesthetic problems such as Zeising’s (1856) golden ratio hypothesis. Yet already in Fechner’s early writings on aesthetics, one encounters a principle which has no direct counterpart among the elements of psychophysics: The Aesthetic Association Principle. By providing the first English translation of Fechner’s (1866) article on *The Aesthetic Association Principle* (*Das Associationsprincip in der Aesthetik*), we hope to raise awareness for an essential aspect of his Aesthetics from Below that has been overlooked: namely, the eminent role of personal recollection, Zeitgeist, and cultural background in the formation of aesthetic experiences. We decided to translate Fechner’s 1866 article because it offers a comprehensive summary of his thoughts on this important matter to him: Based on a public lecture at the Leipziger Kunstverein of the same year, the text was first published in the Zeitschrift für bildende Kunst and later incorporated into the first volume of his *Propaedeutics of Aesthetics* (*Vorschule der Aesthetik*).^2^

Why do people prefer the sight of an orange—after all, an unevenly surfaced and imperfectly shaped object—to that of a perfectly round varnished wooden ball of the same size and colour? Why are red cheeks and lips more attractive than red noses and hands? If the aesthetic appeal of an artwork lies mainly in its formal aspects, should we not value an equally colourful but perfectly symmetrical carpet pattern over Rafael’s Sistine Madonna? It takes but a few casual examples and simple thought experiments for Fechner to demonstrate that aesthetic choices are largely shaped by the observer’s learning history (associative factors) rather than by an object’s size, shape and colour (direct factors). Moreover, since formal properties, such as the colour red, may themselves be evocative of strong associations, both direct and associative factors must be regarded as inextricably intertwined. According to Fechner, it takes an inductive approach to fully grasp the importance of the association principle for aesthetics, which is easily overlooked by the Aesthetics from Above with its “more or less fleeting or floating concepts, that do not capture the individual with the appropriate precision due to their generality” (p. 8). Nevertheless, 150 years after these ideas were first published, they seem to have lost nothing of their relevance as the role of content is still underestimated by today’s paramount theories of empirical aesthetics (Ortlieb & Carbon, 2019). Apart from being highly topical, Fechner’s piercing and evocative line of thoughts impresses with its unsurpassed *Prägnanz.*

## Editorial Conventions

Fechner’s original style of writing is not always easy to read, let alone to translate—beside the fact that his works were mostly published in the German Fraktur typeface which is difficult to read even for German readers of the 21^st^ century. For the sake of conceptual clarity and legibility, we chose a mode of translation that is true to Fechner’s ideas and the overall tone of the text but does not always follow his rather laborious diction. Special attention was paid to a consistent use of terminology. The German “Anschauung”, for example, may translate as “basic sensation”, “perception” or even “strong opinion”, depending on the degree of cognitive elaboration suggested by the context; and when Fechner used the word “Gestalt” he would not have the core concept of Gestalt psychology in mind, which was not yet established at the time, but its meaning in everyday language that translates as “figure” or “shape”. Complete author names and terms of conceptual importance that defy direct translation are given in square brackets: “dependent beauty [*anhängende Schönheit*]” (p. 9). Moreover, page breaks of the original publication are marked in the text by vertical lines with suspended page numbers indicating the beginning of the first complete sentence on a new page. The only footnote from the original publication—a comment by the journal’s editor—is the only one marked with an asterisk, while the translators’ explanatory notes are consecutively numbered. All references and figures have been added by the translators and were not included in the original article. Note: For the interested reader, we provide a digitally remastered facsimile of the original German publication along with the translation.

## Fechner (1866): The Aesthetic Association Principle*--- The Translation^3^

Aesthetics, like many other fields of enquiry, can be treated in two fundamentally different ways, which I will distinguish in the following simply as the Aesthetics from Above [*Aesthetik von Oben*] and the Aesthetics from Below [*Aesthetik von Unten*]. The former works deductively (from general terms to individual ones), the latter inductively (the other way around). The Aesthetics from Above establishes a framework of ideas made up from topmost-ranking aspects, subordinating aesthetic experience to this framework.|^180^ The Aesthetics from Below devises aesthetics entirely from empirical data based on aesthetic experience. The Aesthetics from Above is principally and ultimately concerned with concepts and ideas of beauty, art, style, their position within the overall system of most general concepts, their relationship with truth, with goodness, with the divine—particularly with divine ideas and divine creativity. From the pure heights of these general ideas, one then descends to the level of simple empirical singularities, of specific beauty bound by time and space, evaluating every individual phenomenon with respect to the general. The Aesthetics from Below sets out from singular experiences of what pleases and displeases. From there, it builds up all concepts and laws that have their place in aesthetics, attempting to develop them with regard to the laws of what is and what ought to be—and to these laws pleasure must always be subordinated. By generalizing more and more, we will arrive at a system of the most general concepts and laws. Whereas the Aesthetics from Above focuses on concepts and ideas, with all explanations being merely based on subordinations to categories of concepts or ideas; the Aesthetics from Below focuses on empirical laws, and all explanations are mainly based on subordinations to such.

Each of the two ways has its advantages and disadvantages. Right from the beginning, the Aesthetics from Above provides us with the goal, that the Aesthetics from Below ultimately hopes to attain, namely, the most general view. However, starting from the highest aspect makes it difficult to clarify the causes of pleasure and displeasure in particular cases. Albeit this should also be of our concern, the Aesthetics from Above only offers more or less fleeting or floating concepts that do not capture the individual with the appropriate precision due to their generality. Moreover, this way presupposes a correct starting point only a perfect philosophical or theological system can provide, both of which we still do not have. There is only a great number of attempts at such systems, and accordingly many attempts at tuning aesthetics to these systems, all of them hardly convincing, merely pandering to the urge of having ideas of the highest order and perpetuating this urge. In contrast, the Aesthetics from Below provides clear guidance with regard to the causes of pleasure and displeasure in each individual and concrete case but has difficulties to reach general aspects and ideas; one easily gets stuck halfway, so to speak. Nevertheless, by starting from scratch the Aesthetics from Below does not presuppose anything disputable. It is in this way that we may slowly but surely increase our knowledge about aesthetics.

In short, the relationship between the Aesthetics from Above and the Aesthetics from Below resembles the one between natural philosophy and physics. Just as natural philosophy was there before physics, the Aesthetics from Above preceded the Aesthetics from Below and, up to now, the former remains the beaten path, while the latter has not yet been pursued with determination, consistency, and method. Just like physics will never render natural philosophy obsolete, the Aesthetics from Below will never replace the Aesthetics from Above. Yet I think we are well advised to take the path from below to establish a solid basis for the otherwise unfounded and speculative assumptions of the Aesthetics from Above.

Alas, no more generalities about the two ways. It was only to say as much as to show that the principle to be addressed is both a matter and a test of the Aesthetics from Below. This will also explain why the Aesthetics from Below has received so little attention in the field of aesthetics so far.|^181^ According to the nature of the Aesthetics from Below, it cannot take general aspects as its starting point; thus I will start with the simplest possible examples that allow for the explanation and verification of what I call the Aesthetic Association Principle.

I do not present something entirely new; who could possibly claim novelty in such matters! In psychology, above all, the importance of the association principle has been known and recognised for a long time; even if this is not the case in aesthetics, this principle has not failed to receive some acclaim even there. Occasionally, it is applied by today’s scientifically oriented aesthetics, yet without any notion of its fundamental and far-reaching importance. It was even misinterpreted by [Immanuel] Kant^4^ in his doctrine of dependent beauty [*anhängende Schönheit*]. Among the more recent scholars, only [Hans Christian] Ørsted^5^ has paid more attention to it—comprehensible since he came to aesthetics as an observer—but of course without promoting this principle to its most developed state or to delve into its specifications; and who among the paramount aestheticians attends to Ørsted? Among the older especially Home [David Hume?]^6^ should be mentioned. But let us start with examples!

The most beautiful of all fruits,—or, if one finds the term beauty too exalted—the most pleasurable is probably the orange. In former times, this was even more the case than today, where it is publicly displayed on sales counters everywhere and to be found on almost every lunch table for dessert: Because every stimulus is dulled by its frequency.^7^ But I still lively recall, which, so to speak, romantic charm the sight of this fruit used to have for me and even today one is unlikely to prefer any other with respect to its appearance.

So, what does the pleasure of its appearance consist of? Naturally, everyone immediately thinks of its pure golden colour and its perfectly round shape. And certainly, much lies herein; and maybe one thinks that all its beauty is encompassed herein. Yes, where else should it be? If readers were to ask this, it proves that they are not aware of my principle. For should anything else come to their mind, it would certainly fall under the principle. One may thus think for a moment whether the appeal of this fruit’s appearance truly lies entirely in its beautiful golden colour and perfect roundness!

I say no; for why is it that a yellow varnished wooden ball is not as pleasing as an orange, if we know that it is a ball of wood, not an orange. Indeed, although the orange has a rough skin and roughness is generally less appreciated than smoothness as can be shown by comparing different wooden balls—we still prefer the rough orange to the varnished wooden ball.

This judgement cannot be derived from a favourable form and colour alone, as both objects are about the same regarding both dimensions, so, if at all, the wooden ball should be preferred. A preference for the orange can only be due to the fact that we identify it as an orange, not as a ball of wood, and thereby add the meaning of an orange to its mere shape and colour. Of course, the meaning of an orange is also partly in its shape and colour but not exclusively. It rather lies in the totality of what it is and does, and especially in what it is and does to us personally. Since only shape and colour are immediately present to our senses, memory adds the rest, not as single details, but as an overall impression: It amalgamates with the sensual impression, thus enriching it, illustrating it, so to speak; we might briefly call this the mental colour adjoining to the sensory colour or the associated impression that unites with the direct one.|^182^ This is the reason why the orange appears more beautiful than the yellow ball of wood.

Does someone who perceives an orange, merely see a round yellow patch? With the physical eye, yes; mentally, however, we see an object of delightful smell, refreshing taste, grown on a beautiful tree, in a beautiful country, under a warm sky; we see, so to speak, the whole of Italy along with it, the country, which has always attracted our romantic longing; the mental colour is composed of all these recollections, by which the sensual one is glazed and thus embellished; whereas someone perceiving a yellow wooden ball will only sense dry wood behind the round yellow patch that has been shaped in a turner’s workshop and coloured by a varnisher. In both cases, the impression resulting from memory is immediately associated with sensation, completely merged with it, determining its character so essentially, as if it were part of the pure sensation itself. Only through such comparisons we realise that this is not the case.

Another example:

Why do we find a red cheek on a juvenile face so much more attractive than a pale one? Is it the beauty, the delight of the colour red itself? Undeniably, this has a part in it. A fresh red pleases the eye more than grey or colour errors. We can prove this even by experience. The Englishman [William] Cheselden^8^ did surgery on a person born blind, who had never seen colours and who therefore had not yet acquired any colour-related associations. He declared that scarlet red was the most beautiful of all colours; among the others, he liked the liveliest best, whereas black evoked much discomfort. The savages, who paint their body, prefer to paint it with red colour. The earliest idols out of wood and clay were painted red. So, should we not recognise the natural appeal of the red colour as the simple cause for our pleasure in red cheeks? Again, I ask, why do we not find the same fresh red quite as appealing on the nose and hand as on the cheek? We even dislike it. In the case of nose and hand, the pleasurable impression of the red is obviously outperformed by an element of displeasure. What is the reason for this? It is not difficult to find. The red cheek speaks of health, joy, flourishing life; the red nose reminds us of drunkenness and copper-induced chronical disease [*Kupferkrankheit*], the red hand of washing, scrubbing, mashing; these are things we neither wish to have nor to do. And we also do not wish to be reminded of these things.

Conversely, if a red nose and a pale cheek had always been a sign of good health and temperance, the pale nose and the red cheek would appear as signs for the opposite and the direction of our attraction would also be reversed. North American and Polish women prefer a pale cheek to a red one, and, if necessary, try to obtain a pale one even at the expense of their health by drinking vinegar or by other means. So, is it because they value paleness itself over redness? Certainly not, but because they are accustomed to interpret a pale cheek as a sign of refined constitution, higher education, and social position, while the red one only indicates peasant health, they prefer the first over the latter. It is for the same reason that the Chinese find crippled feet appealing when they belong to their women but evaluate the most natural-beautiful ones vulgar and ungainly. They also depict their deities with big bellies since they are used to seeing big bellies on their most distinguished imperial officials who are above earthly needs and toils that usually stand in the way of big bellies.|^183^

Once I heard a woman say, one could only reliably judge the beauty of a human foot, if it was shod. If sincerity had not been among this woman’s virtues, she most likely would have hesitated to make this statement, as it might appear bizarre to most people. Yet there is some truth in it. When we learn about the meaning of the human foot, it will be hidden by a shoe in almost every case; and hence, we are truly familiar only with the foot inside of a shoe. Our own foot, albeit not always the prettiest, is almost the only one we ever see naked and the feet of statues usually count among their least noted features. Thus, criteria for beautiful feet are less frequently applied to a naked foot than to a shod one; and, while judgement of the former presupposes a certain amount of art expertise, judgement of elegance and daintiness in the case of the latter only requires common social experience.

Not lesser than in the visual domain, the principle is relevant for all other sensory modalities.

A blind woman, who could only acquire shape through the haptic sense, was asked, why the arm of a particular person appealed to her so much. Neither did she feel the gentle pull, the beautiful fullness, nor the elastic swelling of the arm. Actually, it was because she felt that the arm was healthy, lively, and light-weighted. Although she could not feel this directly, she associated it with what she experienced. I do not believe, however, that the direct impression, in which one was inclined to find the sole cause of her appreciation, was really without effect; yet the associated impression was more vividly brought to her attention. In ourselves, the normally sighted, it is the opposite. We believe that we are able to read the entire beauty *off* a beautiful arm, without suspecting that we read most of it *into* the same.

A woman who loved her man very much, said to him: I am so delighted that you have such a pretty name. The name was not very pretty, but she loved the man, that is why she liked the name. I myself remember that when I was a child, I liked the name Kunigunde very much, until I came in contact with a girl of that name with a disagreeable appearance and character. Soon thereafter, the name became disagreeable for me; and as I have not met a particularly amiable Kunigunde since then, this impression has prevailed.^9^

Estate managers usually love the smell of dung because it reminds them of fertility.

Examples of that kind could be extended endlessly.

However, I hear a voice calling down to me from above: What are all these examples good for? What do we gain for the sake of understanding aesthetics, what is gained at all? The orange, the cheek, the nose, the hand, the foot, and so on, are dependent parts of nature and the human body; any aesthetics, however, that does not think low of itself, will go for the whole and account for the parts only as such.

Well then, let us consider the significance of the principle for the beauty of a whole landscape, the whole human figure, a whole work of art, and we will find it not diminished but, on the contrary, extended and increased in the way that the whole surpasses the parts. Using the simplest examples is not only the easiest way to explain the principle, but on our way from below, we also cannot take the direction that appears as the only one possible for the way from above. With a caveat to ascend further in the future, we therefore summarise the principle’s main aspects based on the previous examples.|^184^

Every object we deal with is mentally represented by the resulting impact of our remembering, that is, of anything we have experienced externally or internally, heard, read, thought, learned with regard to this object, and even related objects. This resultant is linked directly to the sight of the object, just as the idea of it is tied to the word that designates it. In fact, shape and colour are, so to speak, nothing but visible words, which immediately and involuntarily bring the whole meaning of an object to our attention; yet, we have to learn this visible language first, to understand it, just like the language of words. When we perceive a table, basically, we only see a rectangular patch; but within this rectangular patch, we perceive everything a table is needed for; this is what actually makes the rectangular patch a table. We perceive a house, but this means everything along with the house, that a house is good for and what takes place in a house; that is what turns the patch into a house. We do not perceive it with the physical eye, but with a mental eye. We hereby do not recall every detail separately that contributes to this impression; how could this be possible, as everything attempts to enter consciousness at the same time. Thereby, everything blends into the intuitive and coherent percept, which we have called the mental colour—a term which is telling in more than one respect. No matter how many different colours we mix, the mixture will always appear as a single colour that changes according to its coloured components, and, if it is transparently applied to a solid coloured background, the two colours together result in a coherent percept. And this percept again directly corresponds to the combination of the two coloured components. Thus, from all the different kinds of remembering, which are linked to an object’s appearance, there results a coherent percept that varies according to the different ingredients in one’s memory, and which also blends into a coherent percept with the physical object’s direct sight. So even in the case of a nearly identical sensory input, a completely different overall impression may arise if it is glazed with different mental colours, only a slight difference in its sensual quality is needed to convey these different links. An orange, a yellow wooden ball, a brass ball, a golden ball, the moon. All of them appear as round yellow patches, and yet what different impressions they convey! We perceive the golden ball with a kind of deep Californian^10^ respect, we imagine entire palaces, chariots and horses, liveried servants and magnificent travels; the wooden ball appears to be for rolling only, but what high ideality belongs to the moon! It is only through this, that anything authentic, the real diamond, the real gold, the real tenuous lace, the real song of the nightingale, gains its tremendously advantageous impression over any kind of imitation, even if it is of most deceiving quality.

According to the premise, that it is essential for our pleasure or displeasure *what* we remember at the moment of remembering or perceiving a certain object, the remembering as such adds a momentum of pleasure or displeasure to the aesthetic impression of an object that may be in accord or discord with other events of remembering and perceiving of the object. This principle will create the most manifold aesthetic conditions which would be worthwhile to examine in examples, but this would lead too far here. The strongest and the most frequent impacts which we receive from an object, in relation to an object and relative to an object, are the ones that leave memories which are the most dominant for subsequent associated impressions.|^185^

Particular remembering is, of course, relatively weak with respect to what it effects as recall; but through accumulation of many such events of remembering and their impact on a single direct impression, their cumulative impact will surpass the effect of the direct impression the more easily, the richer in content they are.

How rich and interesting the remembering of an orange is compared to its mere shape and colour!

An everyday example can teach us, how overpowering an impression resulting from accumulated past experiences may become compared to the sensory, direct impression. Holding one’s finger up in front of the eyes at twice the distance, it appears just as large as before, although its image in the eye is only half the size, and it can only appear half as large to a freshly operated blind person. Resulting from our entire life’s experience, the knowledge that it has the same size at any distance, overpowers the sensory input so completely, that we think to perceive its constant size at every distance. Obviously, our pleasure in regarding objects is far too easily mistaken for the effect of their sensual appearance, whereas this pleasure, in fact, draws on previous experience. Yet it is our mind that adds previously acquired experience to our senses.

I have so far emphasised that the different components blend in the overall aesthetic impression. Yet, for the sake of understanding aesthetics, we have to analyse them in order to account for the formation of the overall impression. We need to ask, what belongs to the direct impression, what is due to associations, and what do the former or latter contribute to it. Such an analysis can never be exhaustive as we cannot calculate the contribution of our remembering to every single associative impression, in fact our associations are a kind of echo of our entire life, with different weightings of its various moments. If we hit a taut fabric in some spot—our imagination is comparable to such a fabric—the whole fabric will vibrate, but especially those parts that are closest to the spot we have hit or that are connected with it by the strongest threads. However, an impression will always hit our mental fabric simultaneously in more than one spot. Even if all our mental properties resonate with every single impression, we are still able to examine the predominant aspects of each impression. Such considerations are alien to today’s standard theories of aesthetics as they prefer to ignore the question altogether.

Aesthetics would be well advised to examine the composition of an overall impression. Although the overall percept cannot be described, we may be able to characterise the combination of its various components. Who could possibly define the percept of an orange, a gold ball or a wooden ball? It can, however, be described through the associations which have merged to shape it.

Not only through those that have merged into it, but also through those that may arise from it, which constitutes a new, important aspect. Indeed, any association that has contributed to a mental impression may come to the foreground again; it only takes external or internal occasions for this to happen. This is why, after obtaining an overall percept, the subject matter may be examined more closely in different, yet interrelated directions, forming a second main component of an object’s aesthetic effect, that does not arise solely from its coherent overall percept.|^186^ It is like the seed that produces a plant that resembles the one from which it came. At the same time, this result of remembering is the fountain from which phantasy draws; and since beauty has in our times been explained entirely with recourse to phantasy, this is an invitation to examine this source more closely than hitherto.

By emphasizing the mental colour of things, one should not overdo it, though this might be tempting, after one has come to understand its importance. Just imagine an orange of a grey inconspicuous colour instead of a beautiful golden yellow with a skewed and crippled form instead of a perfectly round one, no memory attached to it will ever render it beautiful or pleasant. A vanilla bean evokes similar rememberings as an orange but who would therefore find it good-looking! Yet again we should not underestimate the power of associated impressions. The comparison between the orange and the wooden ball indicates this. Neither the direct nor the associated impression accomplish anything by themselves, but conjointly they accomplish much, together they add up to more than a mere additional product of their pleasures. Here again, the very general and far-reaching principle of Aesthetic Amplification^11^ takes effect—yet another principle that is not recognised by the Aesthetics from Above. Although it is very important to explain how the direct sensory input and the associated impression interact to form a coherent percept, we cannot go into further details at this point.

These are the most general aspects of the principle, which can be clarified by the simplest examples, and they remain equally valid when we now apply it to examples on a higher level.

Let us try to account for the percept the sight of a landscape evokes! There is something unspeakable in it, something that cannot be described exhaustively. How can the nature and the causes of this impression be explained? To show the different explanations given by the Aesthetics from Above versus the Aesthetics from Below, I will contrast both approaches by taking an explanation from one of today’s most valued textbooks on aesthetics by [Moriz] Carrière.^12^ The first is the most far-reaching explanation; it is linked to the highest of idealist aspects, while our explanation is found in closest proximity and linked to the lowest ones.

Carrière says (p. 243):

The essence of nature itself corresponds to beauty; for beauty appears to the mind as a manifest representation of ideal content and mental regularities. This is what delights us so deeply, when our mind meets some affinity with our soul in the external and physical world. First and foremost, however, one’s individual life is the purpose of life in general, every being is there for its own end and not created to please us with its appearance; it is a favour of fate, if the totality of the universe presents the reciprocity of things, the how and why of their mutual relations in a way that we may perceive and grasp the inner essence from the surface as it appears from our limited point of view, and how the forms of things answer not only to the universe’s ultimate purpose, but also to the conditions and demands of our individuality. Indeed, we may praise therein the benevolence and magnificence of the world’s ultimate cause, when substances, that appear indifferent to the life of the organism, namely of the plants, or that are exhaled by them, delight us with their pleasant smell or radiant colour like etheric oils or pigments, etc..|^187^

And in order to show how the individual is accounted for by this general consideration, Carrière informs us about the plant as an element of the landscape (p. 258):

The potencies of the inorganic nature are focussed in the plant, in that an individual idea comes into effect as a body-forming life force [*leibgestaltende Lebenskraft*] actively reproducing the organism over and over again, that is connected with the earth by its roots, yet rises into air and light spreading its branches and leaves sideways. The plant illustrates the concept of organic design, which we have claimed for beauty earlier: the diversity of leaves and branches emanates from a unity and is manifestly supported by it, and the interaction of the individual figures forms a harmonious whole.

Admittedly, our perspective from below does not come up to this grandiose vision. Let us rather stick to what we can do better and consider the following simple example.

To the eye of the blind-born person who has just successfully undergone surgery and who looks outside for the first time, the entire nature initially appears as a marbled page because the patient is not yet able to perceive and simultaneously grasp the meaning of the perceived. When gazing into the distance, there are meadows, fields, woods, mountains and lakes, but these meadows, fields, woods, mountains and lakes cannot be deciphered; there are only green, yellow, light, dark patches to be seen. Only the feeling of a far-reaching gaze, the sensual stimulus or, going slightly beyond the sensory input, the sensation of light and darkness, colour contrast, variation, and change determine the impression which can be received from the landscape. But is that really all information we can retrieve from a perceived landscape? We, too, see all that; it certainly adds considerably to the impression a landscape makes, the mood it evokes; yet in the distant woods, which is but a green patch to the unexperienced eye, we also perceive something vividly thriving and growing, that provides shade, coolness, wherein the hare, the deer run, the hunter walks, the birds sing, that is haunted by some fairy tale, although we do not really hear or see any of this. In the lake, that is just a blank or a blue patch to the patient, we know, waves go, skies are reflected, fish play, ships cruise, and so on. Associations arise from all of these things that thrive and grow and undulate. With our physical eye, we do not see the woods and the lake any different than the freshly operated blind person and the new-born child: green and blank or blue patches; but everything we have ever heard, seen, read, experienced, thought with regard to woods and lakes, like anything that may serve as a comparison, contributes to the associations these objects bestow upon us. This turns the sight of them into something unspeakably more meaningful, rich, lively, emotionally deep and more productive for the imagination, than for the person who has not seen, heard, thought about them. And the way it is with woods and lakes, it is with every element of a landscape, meadow, field, mountain and house. Everything is linked to our remembering and to notions of comparison, whereby these objects become meaningful to us, and likewise their combination acquires meaning. The sum of these memories and associations now merges with the sensory foundation and its internal relations to generate the general percept of the landscape; with every detail of the landscape opening a different field of memories and associations; and whatever enters into it might as well emerge from it.|^188^

**Figure 1:**
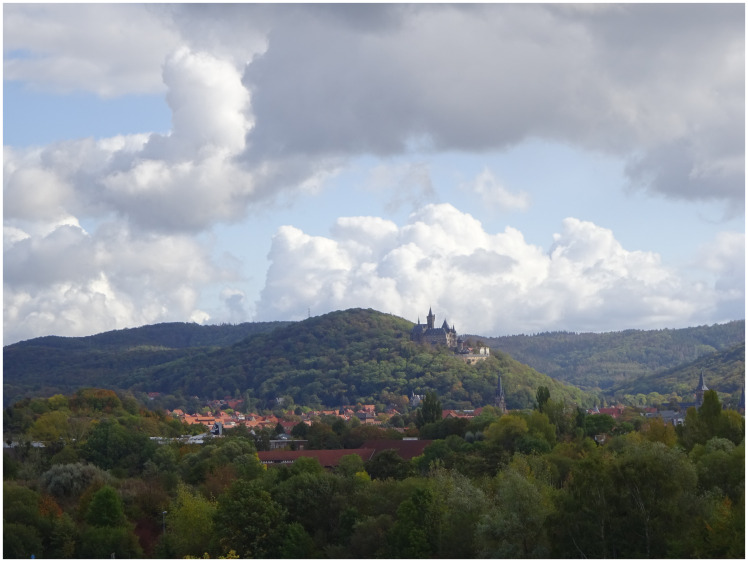
The city and castle of Wernigerode seen against the backdrop of the Harz mountains. Photo by Barbara Ortlieb (2019).

**Figure 2:**
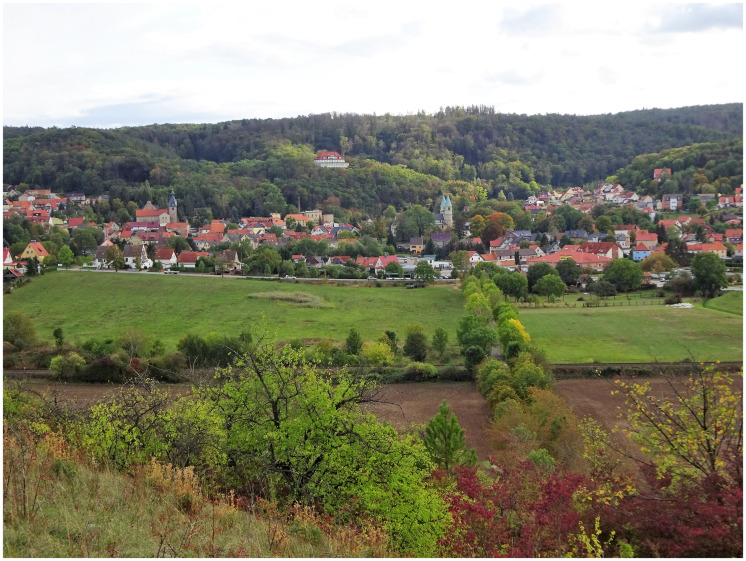
Village of Gernrode at the foot of the Stubenberg. Photo by Barbara Ortlieb (2019).

**Figure 3:**
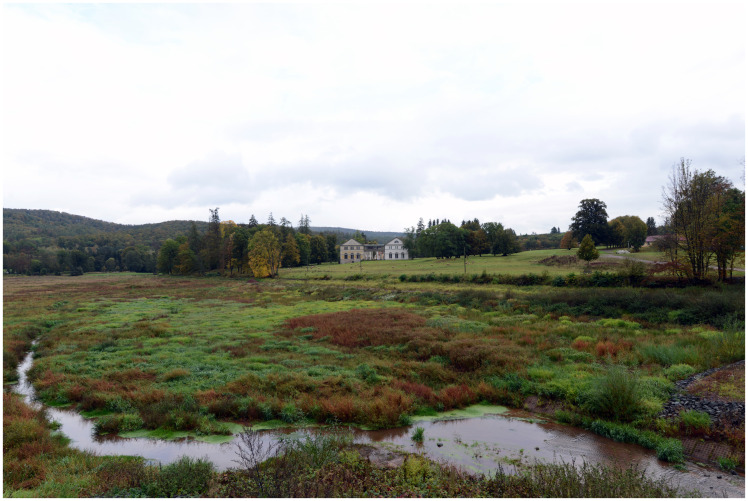
Castle and park of Wilhelmsthal in the Thuringian Forest near the city of Eisenach. Photo by Barbara Ortlieb (2019).

This said, it is easily understood, where the unspeakable, inexhaustible, inexplicable of a landscape’s impression comes from. Who could possibly pursue, exhaust and clarify all the ideas that have contributed to it? In this respect, every single object is inexhaustible; the landscape, so to speak, provides us with an inexhaustible variety of inexhaustible objects with infinitely intertwining fields of associations. Yet, even here we are able to identify its predominant elements and thus to characterise, clarify and explain the impression at least to a certain degree. For example:

Everybody will have noticed, what effect, what meaning an otherwise insignificant scenery receives from a properly placed house, a castle and a little village; one might think of the castle of Wernigerode (see Figure 1), the village of Gernrode at the foot of the Stubenberg (see Figure 2) and the houses down in Wilhelmsthal (see Figure 3).^13^ To imagine the scenery of these places without their man-made elements is to rob them of their point. To a certain extent, the delight of this composition of architecture and nature can be gained from the combination, change and opposition of its different shapes and colours; alas, how very little would this signify without any reference to the meaning attached to this combination, this change, this opposition! The main effect arises from the remembering of the human condition, the relationship of human and nature, which appears partly as a domination of nature, partly as an opposition to nature, partly as an interaction and life with and within nature, which joins and enriches the direct impression.

One must not say, although it has been said to me: All of this would also be present in our imagination without the sight of the building in nature, apart from the scenic impression; hence, this impression cannot be based on such associations.—But, while it takes deliberate effort to imagine all of the details separately, successively, incompletely and without any connecting, unifying tie, they are immediately bestowed upon us at the sight of the building in nature as part of the overall impression. These are obviously two completely different things that may result in very different impressions.

To this effect, I would like to give an example from my own experience, where all of this occurred to me:

During my last holidays, my wife and I spent several weeks in a forester’s lodge about fifteen minutes from the city of Lauterberg in the Harz mountains. On the opposite side of our dwelling, there was a green hillside, which we often climbed, and from where we overlooked a vast forested and mountainous scenery of rather indefinite forms. Except for the lodge in the foreground, there were no other human dwellings to be seen anywhere; only in the distance a single red roof protruding from the monotony of the ramps of the greenwoods. Yet this roof brought a very peculiar effect into the otherwise plain scenery. It was simply the punchline of the entire landscape. And I said to myself: What if one made a red spot of the exact same measurements onto a green wall, would it look just as idyllic, sentimental, romantic, fabulous as the red roof in the forest landscape? Certainly not. And could the red spot on the green wall even evoke the same lively associations of man’s living and moving, the sorrows and joys of forest solitude, just like the red roof in the woods does?|^189^

Yet again, I have to mention an objection someone expressed regarding this example, someone who was educated in the new school of aesthetics and would not tolerate the introduction of a new godhead for which he took the Association Principle.

Everything that remembering has added to the impression of the red roof and the green woods, he argued, everything that came into play only through secondary associations, does not belong to the aesthetic impression of the landscape at all, but has to be separated from it in order to be purely aesthetic. For the mere scenic impression, the artist is interested in the, so to speak, musical relations of colour and shape. And these relations affect us directly through the eye and we complete the truly visible, for instance the roof as a part of the house and the green forest representing the woods, by our imagination. Only what is immediately visible of the house and the woods, and thus interacts with other visual relations, is of importance for their scenic impression.

However, this objection is based on the delusion that the mere visual properties of a house and a forest are much more than just meaningless lines filled with colour whose interactions with other visual relations are also without meaning. Only the expedience of the house for living and the tree’s inherent potential for growth and what is attached to these two features brings content, life and depth into the impression of their visible aspects; and to subtract it would be like stripping the flesh from the human body and taking the skeleton for its essential meaning. How might this objection account for the scenic impression of a ruin? Should this impression rather be dependent on the contrast between its grey shapelessness and the colours and lines of its surroundings; or rather on what is now the ruin as a focal point of memories in the vivid present; it would otherwise take but the grey rugged rock without a ruin on top to make the same impression. How could we possibly speak of a landscape’s romantic, idyllic, historical character if the higher artistic significance of the contrasting, harmonic and rhythmic relations of the colours and forms were not provided by the meaning these visible relations have acquired over a person’s lifetime. As far as these features are concerned, they only gain higher landscape-related significance as part of such higher level relations; as bearers of higher level aspects their impact is then, of course, intensified in accordance with the Amplification Principle. But let us postpone the dispute of this matter for now and mention some other experiences instead.

Nowadays, many castles and monasteries on hills and mountains serve as madhouses and prisons; once we learn about this, it seems as if the charm they bestow upon the landscape is put out by a splurge of cold water.^14^

Today’s railway buildings count among the greatest achievements of contemporary architecture. Such grandiose and characteristic structures of the purest architectural symmetry can be seen in many places! Moreover, they can show the perfect functionality, and who would deny the importance of functionality for the aesthetics of architecture, which is basically also conveyed by association? Yet the impression of these buildings is never delightful to the ultimate degree and falls short of the highest esteem; they never grant the joyful impression of a palace nor the sublimity of a temple.|^190^ Why? Because we identify them as theatres of a displeasing hustle and bustle.

Let us go even higher and beyond landscape and architecture! We recognise the human figure^15^ [*Gestalt*] as the most beautiful work of creation; the highest works of fine art are dedicated to it or use it as their elements. Doubtless, there is much in the flowing of shapes, the mirror-symmetry, maybe the simple proportions, as some claim, or certain rhythmical relations as others have it, or the golden section as [Adolf] Zeising^16^ asserts, and certainly also something instinct-driven that is appealing about the individual figure, apart from any meaning related to it; besides, the painting as a whole also comprises the aspects of grouping and colour treatment in which harmonic and disharmonic relations in themselves may also bear a part. However, all this is but the lowest foundation for the human figure as an expression of functionality for the affairs and joys of life and the higher expressions of the soul and its movements which we find entirely within a single figure and which are ultimately surpassed by the more general and higher human relations, yes, even relations transcending the human sphere, we find in the painting as a whole. All of this, however, bears on the perceived constellations of shape and colour only through the meaning they have acquired due to our previous experiences; all of this is a matter of the associated, not the direct impression. One must not despise these basic foundations of human beauty, just as one must not disregard metre, rhythm and rhyme in poetry; but we must not value them too high either; and who can search for the highest beauty in metre, rhythm and rhyme of a poem, if their violation spoils the entire beauty of a poem just as much as a perfect flow can lift it up? We have here yet another example of the Aesthetic Amplification Principle whereby the product of lower and higher factors, which rest upon the former, may result in a higher aesthetic pleasure than the sum of aesthetic pleasure which lies in every single one of the lower and higher elements. In this regard, there is no difference between the beauty of a poem and that of the human body.

Many aesthetic theories acknowledge how a building’s functionality contributes to its beauty just as the mental expression adds to the beauty of a human being. These theories approve of the Association Principle, albeit it is not explicitly credited and its most important aspects are not recognised. Often the mental expression is identified with the direct impression as a predefined or automatically emerging appendage of shape. Often the impact of association is confounded with direct impression; and its essential contribution to higher aesthetic impressions is often, as in one of the previous examples, fundamentally misunderstood, even denied; equally often beauty is also entirely attributed to functionality and meaning without any good reason. Much could be said about all this, but on the whole, we have promised to refrain from treading the path which we do not deem the best. We therefore close as we began: with some general reflections.

Should the appeal of things be based mainly on remembering pleasant things, some things must be pleasurable *per se*. It is one of the main tasks of the Aesthetics from Below to detect these sources of inherent delight and appeal as well as the laws of their interplay and possibly to identify a general source, a general law of how pleasure and displeasure come to pass, which is a hitherto unsolved problem like the problem of a universal law of energy in physics.^17^ However, we did not attempt to solve such general problems. We only meant to show that association is one of the most important among the secondary sources of appeal in that it absorbs the more direct ones and blends them.|^191^

One must not mistake the direct sources of pleasure for the purely sensory ones alone, although all of the purely sensory are direct ones. Conversely, higher aesthetic impressions are not limited to the field of association: Perception of symmetry, colour relations, relations of tones, goes beyond the purely sensory domain without therefore being a matter of association.^18^ All higher aesthetic impressions are attached to contexts and interrelations that can be internal as well as external to the object. Here the internal, there the external or associative ones play the predominant role.

In the visual modality, there is no aesthetic impression of considerable height which does not involve the Association Principle. Kaleidoscopic figures and fireworks are the highest the visual domain brings about without it. The experience of poetry also culminates in the associative factor, for the meaning of a poem is linked to the words, whereas meter, rhythm, rhyme only gain significance by merging into them. In music, however, association only plays a supporting role. The higher impression of music lies mainly in the pursuit of its internal relations and whatever else is linked to the musical impression is associated to it by coincidence.^19^ Striving for the establishment of consistent principles, the overall impression of painting has also been frequently linked to internal relations like the ones found in music; but in this respect, painting is more akin to poetry than to music, although they are not in every respect comparable. It would be of some interest to examine the partly analogous and partly differing role of the Association Principle in the different arts more closely; yet again, we have to remember the limits that are imposed upon us here.

For two reasons, beauty itself does not become a matter of pure chance just because it is largely based on incidental associations. First, around every object a certain circle of associations necessarily arises from innate dispositions and the relationships among people and things that are based on them; second, among those which vary according to different conditions in time and space, only a particular form is particularly benign for mankind and this is the one that is linked to the concept of true beauty. This, of course, necessarily follows from our principle and it can be easily held against the idea of absolute beauty some people have, that beauty of the human figure cannot be of the highest visual beauty everywhere. If there are creatures on other planetary spheres that are organised and built differently from humans on account of other cosmic conditions, the highest beauty will be attributed to those creatures with the most valued meaning attached to them.

For humans, beauty is essentially based on associations that relate life’s basic sensory sensations to things of higher value and meaning; this is why beauty powerfully affects all higher cultural relations of mankind just as much as beauty is in a way their product and thereby gains a high significance that goes far beyond immediate pleasure.

Translated from the German of Fechner (1866) by S. A. Ortlieb, W. A. Kügel and CCC.

## Supplemental Material

sj-pdf-1-ipe-10.1177_2041669520920309 - Supplemental material for Fechner (1866): The Aesthetic Association Principle—A Commented Translation Click here for additional data file. Supplemental material, sj-pdf-1-ipe-10.1177_2041669520920309 for Fechner (1866): The Aesthetic Association Principle—A Commented Translation by Stefan A. Ortlieb, Werner A. Kügel Claus-Christian Carbon in i-Perception

## Note to the Original Paper

* In the original article, the title is accompanied by the following editorial note: “Thus far, the program of our journal did not include aesthetic treatises; yet we believe that it is in the very interest of the readers if we admit this original attempt ‘to introduce a new god to aesthetics’, especially since it was already well received as a public lecture”.

## Notes

1 Apart from the first volume of the *Elements of Psychophysics* (Fechner, 1860/1966), there are only two other English translations: Fechner’s (1836/1906) popular booklet *On Life After Death* and a compilation of his philosophical writings entitled *Religion of a Scientist* (Fechner, 1946).

2 The German “Vorschule” literally translates to “preschool”. From Fechner’s (1876) introductory remarks on the title, we conclude that he used it in the sense of “propaedeutics” like other authors before him (e.g., Paul, 1804). Thus, “Vorschule” refers to an introductory course offering the basic knowledge and skills needed to conduct studies in a particular field, in this case (empirical) aesthetics.

3 Original publication: Fechner, G. T. (1866). Das Associationsprincip in der Aesthetik. *Zeitschrift für bildende Kunst, 1*(1), 179–191. Page breaks of the original article are indicated by short vertical lines with suspended page numbers. They mark the beginning of the first complete sentence on a new page.

4 Philosopher Immanuel Kant (1724–1804) whose 1790 *Critique of Judgement* (*Kritik der Urteilskraft*) proved tremendously influential in Western aesthetics. Fechner (1876) and later Bourdieu (1979) held the Kantian ideal of *free beauty* (*freie Schönheit*) responsible for a highbrow aesthetics that values style over content and looks down on popular taste. Ortlieb and Carbon (2019) have made a case that, against Fechner’s original intentions, a highbrowed disregard for content-related associations and everyday phenomena is still present in today’s paramount models of empirical aesthetics, including fluency-based ones.

5 In 1796, physicist and chemist Hans Christian Ørsted (1777–1851), who later became famous for his discovery of electromagnetism, authored an award-winning essay in aesthetics about the “boundaries between poetic and prosaic expression” (Ørsted, quoted by Christensen, 2013, p. 48). His *Dissertation on the Structure of the Elementary Metaphysics of External Nature* (Ørsted, 1799), however, was based mainly on the works of Kant (Wilson, 2008).

6 By the name “Home”, Fechner most likely refers to philosopher David Hume (1711–1769; born David Home—sic!) whose ideas about aesthetics proved just as influential (*Of Tragedy*; *Of the Standard of Taste*, both published in 1757) as his radical take on the problem of induction. Especially, Hume’s (1757a,1757b) much-quoted claim that “[b]eauty is no quality in things themselves [but] exists merely in the mind which contemplates them; and [that] each mind perceives a different beauty” (p. 136) is widely agreed with the ideas Fechner develops in the present text about the Aesthetic Association Principle.

7 On the whole, Fechner takes a very differentiated look at the role of familiarity in aesthetics: Although he rejects the idea of a monotonously increasing relationship between familiarity and liking (see Zajonc, 1968), his many examples, especially the below passage on remembering, indicate, that he is perfectly aware of the fact that familiarity, that is, previous exposure, is crucial for the establishment of context- and content-related associations. Fechner even accounts for the exceptions (preparedness; Seligman, 1971): things that make a strong first impression which we are today inclined to identify as phenomena like the baby scheme or images of snakes and spiders that reliably trigger affective responses without or with only very little previous experience (see Hoehl et al., 2017).

8 William Cheselden (1688–1752) was an anatomy teacher and surgeon who performed one of the first known cases of full recovery from blindness—a 13-year-old boy—in 1728. Although the patient gained sight after removing the opaque lenses caused by a congenital cataract, he encountered severe problems even with simple visual perception tasks (Gregory & Wallace, 1963).

9 By contrast, in the two translators from the University of Bamberg, the name Kunigunde evokes mainly positive associations since the city of Bamberg was an important residence and burial site of the Holy Roman empress and Catholic saint Kunigunde of Luxembourg (c. 975–1040). Apparently, associations are shaped by one’s previous experience. With the Aesthetic Association Principle a high degree of subjectivity comes into play, which is possibly one of the reasons why experimental aesthetics is so reluctant to admit its great importance for aesthetic experience.

10 Perhaps an allusion to the Californian gold rush which came to an end only 10 years before the article was published. Fechner could therefore assume that gold and California (still nicknamed “The Golden State”) were strongly linked in the minds of his readers.

11 Fechner’s (1876) Aesthetic Principle of Help and Amplification (Princip der ästhetischen Hülfe und Steigerung—*sic*!) postulates that “a conjuncture of multiple, alone rather ineffective but altogether consistent pleasure-related conditions yields a hedonic result that is greater, often far greater, than the sum of their individual effects; it is possibly through such a confluence that they exceed the hedonic threshold and yield aesthetic pleasure in the first place” (p. 51, transl. by S. Ortlieb). The success formula for kitsch, proposed by Ortlieb and Carbon (2019), makes as good example for this principle in that it shows how two rather insignificant variables—an immediately identifiable and perfectly conventional manner of representation—multiply the impact of an emotionally charged subject matter.

12 Moriz Philip Carrière (1817–1895) was a writer and philosopher. Fechner is quoting the first volume of Carrière's (1859) *Aesthetics: The Idea of Beauty and its Realisation in Life and Art* (*Aesthetik: Die Idee des Schönen und Ihre Verwirklichung im Leben und in der Kunst*).

13 The city of Wernigerode and the village of Gernrode are both located in Saxony-Anhalt on the northern edge of the Harz mountains, while Wilhelmsthal refers to a secluded castle situated in the Thuringian Forest near the city of Eisenach. All three points of Fechner’s interest make popular postcard motives although visitors, including the first translator and his wife, tend to agree that it is not so much the topography of these places, but the castles of Wernigerode and Wilhelmsthal, respectively, the Romanesque Saint Cyriacus Church of Gernrode, that make them truly picturesque.

14 This could be a hint at the cold-water cures for certain mental illnesses in 19^th^-century psychiatric institutions.

15 Although Fechner uses the term “Gestalt” in this context, it is rather improbable that he is referring to the core concept of Gestalt psychology which was only fully developed decades later. Most certainly, he had the colloquial German word “Gestalt” in mind which closely corresponds to the English “figure” or “shape”.

16 Adolf Zeising (1810–1876) claimed that the golden section is a universal principle, discernible not only in the arts (harmony in music, painting, sculpture, and architecture), but in almost every animate (proportions in plants, animals, and humans; *Neue Lehre von den Proportionen des menschlichen Körpers*, Zeising, 1854) or inanimate natural phenomenon (geometry of crystals and chemicals; *Das Normalverhältniss der chemischen und morphologischen Proportionen*, Zeising, 1856). Although Fechner (1865,1871) did not share this universal claim, he recognised the relevance of the golden ratio for aesthetics and conducted several groundbreaking albeit inconclusive experiments on Zeising’s golden section hypothesis with regard to aesthetic choices (*Ueber die Fragen des goldenen Schnittes; Zur experimentalen Aesthetik*).

17 Written only shortly after Darwin’s (1859) *On the Origin of Species*, this passage seems to anticipate a basic idea of Darwinian aesthetics, namely, to identify universal aesthetic preferences that strongly suggest ancient psychological adaptations (Dutton, 2009).

18 Interestingly, Fechner is speculating about a low-level preformation of basic sensations that already affects aesthetic choices prior to content-related interpretation. This idea is strikingly similar to the concept of a “good Gestalt” that was developed decades later by Gestalt psychology.

19 Fechner seems to adhere to a rather narrow understanding of music in the sense of absolute music, which does not include the possibility of program music, where music is used to illustrate or highlight certain historical events—Wellington’s Victory by Beethoven—or impressions of nature—Beethoven’s 1808 symphony No. 6 (Pastoral). In these cases, it seems that music is very well capable of conveying clear-cut impressions of a battle or a thunderstorm. Fechner’s notion is also challenged by the music drama of his contemporary, the composer Richard Wagner (1813–1883), who made elaborate use of short signature sequences to identify different characters, ideas, objects or places of his music dramas. Through this technique of leitmotifs—Wagner himself spoke of “Gefühlswegweiser” (signposts of emotion)—he was able to create a complex musical commentary to the plot, guiding the listener’s memories and associations. Today, this narrative use of musical themes and leitmotifs is an important feature of film scores.
