# Supplementary material for: Fechner (1866): The Aesthetic Association Principle—A Commented Translation
Source: Iperception. 2020 May 21;11(3):2041669520920309. doi: 10.1177/2041669520920309 (PMC7264472; doi:10.1177/2041669520920309)
Supplement: sj-pdf-1-ipe-10.1177_2041669520920309 - Supplemental material for Fechner (1866): The Aesthetic Association Principle—A Commented Translation [file sj-pdf-1-ipe-10.1177_2041669520920309.pdf]

Das Associationsprincip in der Aesthetik.*)

Von

G. Th. Fechner.

Die Aesthetik läßt sich, wie so manche andere Lehren, auf zwei grundverschiedene Weisen behandeln, welche ich kurz als eine Behandlung von Oben herab und von Unten herauf unterscheiden will. Man behandelt sie von Oben herab, indem man von allgemeinsten Ideen und Begriffen ausgehend zum Einzelnen absteigt, von Unten herauf, indem man vom Einzelnen zum Allgemeinen aufsteigt. Dort ordnet man das ästhetische Erfahrungsgebiet einem von obersten Gesichtspunkten aus konstruirten ideellen Rahmen nur

*) Obwohl wir ästhetischen Abhandlungen in dem Programm unserer Zeitschrift keinen Raum geboten haben, glauben wir doch diesen originellen Versuch „eine neue Gottheit in die Aesthetik einzuführen" im Interesse der Leser bereitwillig Raum bieten zu sollen, um so mehr als derselbe bereits in Form einer populären Vorlesung sich des Beifalls zu erfreuen hatte. A. d. H.

ein und unter; hier baut man die ganze Aesthetik auf Grund ästhetischer Erfahrungen von unten an auf. Dort handelt es sich in erster und zugleich höchster Instanz um die Begriffe und Ideen der Schönheit, der Kunst, des Stils, um ihre Stellung im Gesammtsystem allgemeinster Begriffe, ihre Beziehung zum Wahren, zum Guten, zum Göttlichen, insbesondere zu den göttlichen Ideen und der göttlichen Schöpferthätigkeit. Aus der reinen Höhe dieser Allgemeinheiten steigt man dann in das irdisch-empirische Gebiet des einzelnen, des zeitlich und örtlich Schönen herab, und mißt alles Einzelne am Maßstabe des Allgemeinen. Hier geht man von Erfahrungen über das, was gefällt und mißfällt, aus, stützt hierauf alle Begriffe und Gesetze, die in der Aesthetik Platz zu greifen haben, sucht sie unter Mitrücksicht auf die Gesetze des Seins und Sollens, denen die des Gefallens immer untergeordnet bleiben müssen, mehr und mehr zu verallgemeinern und dadurch zu einem System allgemeinster Begriffe und Gesetze zu gelangen. Dort liegt das Hauptgewicht überhaupt auf Begriffen und Ideen, und alle Erklärungen beruhen nur in Unterordnungen unter begriffliche und ideelle Kategorien; hier liegt das Hauptgewicht auf erfahrungsmäßigen Gesetzen, und die Erklärungen beruhen in der Hauptsache auf der Unterordnung unter solche.

Jeder von beiden Wegen hat seine Vortheile und seine Nachtheile. Der erste giebt uns von vorn herein das Ziel, dem man auf dem zweiten erst zustreben muß, den allgemeinsten Blick, die höchsten Gesichtspunkte; aber man gelangt auf ihm schwer zu einer klaren Orientirung über die Gründe des Gefallens und Mißfallens im Einzelnen, um die es uns doch auch zu thun sein muß; es bleibt mehr oder weniger bei unbestimmt schwebenden, in ihrer Allgemeinheit das Einzelne nicht leicht scharf treffenden Begriffen. Dazu setzt dieser Weg, um richtig zu führen, einen richtigen Ausgang voraus, den man im Grunde nur in einem vollendeten philosophischen und selbst theologischen System finden kann, was wir beides noch nicht haben. Nur viele Versuche derselben haben wir, und so haben wir auch viele Versuche, die Aesthetik damit in Beziehung zu setzen, die alle noch viel zu wünschen übrig lassen, aber doch dem Bedürfniß allgemeinster und höchster Gesichtspunkte entgegenkommen, und, wenn sie dasselbe nicht vollständig befriedigen, doch beschäftigen und wach erhalten. Der zweite Weg gewährt hingegen eine klare Orientirung über die Gründe des Gefallens und Mißfallens im Einzelnen und Nächsten; aber man gelangt auf ihm schwer zu allgemeinsten Gesichtspunkten und Ideen; man bleibt so zu sagen, leicht auf halbem Wege stecken. Er setzt nichts Bestreitbares voraus, fängt aber auch so zu sagen mit nichts an; man schreitet auf ihm sicher in der ästhetischen Erkenntniß vor, aber nur, indem man langsam vorschreitet.

Kurz der erste Weg verhält sich zum zweiten, wie sich die Naturphilosophie zur Physik verhält. Wie nun die Naturphilosophie früher dagewesen ist als die Physik, so ist der erste Weg in der Aesthetik früher dagewesen als der zweite, und selbst jetzt noch der fast allein betretene; wenigstens wurde der zweite niemals mit Entschiedenheit, Konsequenz und Methode verfolgt. Nun wird die Physik niemals eine Naturphilosophie und der zweite Weg in der Aesthetik niemals den ersten überflüssig machen; es würde aber meines Erachtens gut sein, wenn man auf diesem zweiten Wege, dem Wege von Unten, dem ersten, dem Wege von Oben, mehr entgegenkäme, als seither der Fall, um einen festen und sichern Boden als Unterbau für die sonst nur zu schwankende und haltlose Höhe des ersten zu gewinnen.

Genug der Allgemeinheiten über beide Wege. Es galt nur, so viel hier darüber zu sagen, um das Princip, von dem die Rede sein soll, als eine Sache zugleich und Probe des zweiten Weges hinzustellen, und damit zu erklären, warum es in der seitherigen

Aesthetik so wenig Beachtung gefunden hat, — es liegt eben nicht auf ihrem Wege, — endlich auch, um damit den im Folgenden einzuschlagenden Gang der Betrachtung zu motiviren. Nach der Natur des zweiten Weges kann derselbe nicht mit allgemeinen Gesichtspunkten anheben; also werde ich vielmehr mit den einfachst-möglichen Beispielen beginnen, an denen sich das von mir sogenannte ästhetische Associationsprincip zugleich erläutern und bewähren läßt.

Damit behaupte ich nicht, etwas absolut Neues vorzutragen; wer könnte das überhaupt in solchen Dingen! Zuvörderst ist das Associationsprincip nach seiner Wichtigkeit in der Psychologie längst bekannt und anerkannt; und wenn dieß in der Aesthetik keineswegs entsprechend der Fall ist, so hat es doch nicht verfehlen können, sich auch hier irgendwie geltend zu machen. Gelegentlich wird es ja wohl auch in der heutigen Fach-Aesthetik zur Erklärung zugezogen, nur ohne Ahnung seiner principiellen weittragenden Wichtigkeit, die sogar von Kant in seiner Lehre von der anhängenden Schönheit geradezu mißkannt ist. Von Neueren wüßte ich überhaupt nur etwa Oersted zu nennen, der ihm etwas größere Beachtung geschenkt — erklärlich, weil er als Beobachter zur Aesthetik gekommen, — ohne freilich dem Princip zu seinem ganzen Recht zu helfen oder in seine Bestimmungen genauer einzugehen; und wer von den tonangebenden Aesthetikern kümmert sich um Oersted? Von Aelteren möchte insbesondere Home anzuführen sein. Doch beginnen wir mit den Beispielen!

Unter allen Früchten vielleicht die schönste, oder, wenn man den Ausdruck schön zu viel findet, für das Auge reizendste dürfte die Orange oder Apfelsine sein. Früher war dieß sogar noch mehr als jetzt der Fall, wo sie sich auf allen öffentlichen Verkaufstischen ausgelegt, bei fast jeder Mittagstafel zum Dessert findet: denn jeder Reiz stumpft sich durch seine Häufigkeit ab. Ich erinnere mich aber wohl, welchen so zu sagen romantischen Reiz der Anblick dieser Frucht früher für mich hatte und noch jetzt dürfte man ihr keine im Aussehen vorziehen.

Worin nun liegt das Reizende ihres Aussehens? Natürlich denkt jeder zunächst an ihre schöne reine Goldfarbe und reine Rundung. Und gewiß liegt viel hierin; vielleicht meint man sogar, daß Alles hierin liege. Ja, worin sollte es denn sonst liegen? Aber, wenn der Leser so fragte, so wäre dieß ein Beweis, daß ihm mein Princip nicht präsent ist, oder sollte ihm noch etwas beifallen, so würde es sicher unter das Princip treten. Also möge man einen Moment überlegen, ob wirklich der ganze Reiz des Aussehens dieser Frucht in ihrer schönen Goldfarbe und reinen Rundung begründet ist!

Ich sage nein; denn warum gefiele uns nicht sonst eine gelb überfirnißte Holzkugel ebenso gut wie die Orange, wenn wir wissen, daß sie vielmehr eine Holzkugel als eine Orange ist. Ja, trotzdem, daß die Orange eine rauhe Schale hat und Rauhigkeit im Allgemeinen minder gut gefällt als Glätte, wie sich beim Vergleich verschiedener Holzkugeln selbst beweist, so gefällt uns doch die rauhe Orange besser als die lackirte Holzkugel.

Das kann nicht in einem Vorzuge der Wohlgefälligkeit der Form und Farbe an sich selber liegen; in dieser Hinsicht sind sich beide Gegenstände gleich, oder kann die Holzkugel selbst den Vorzug haben. Der Vorzug der Orange kann nur darin liegen, daß wir eben eine Orange, aber keine Holzkugel in ihr sehen, daß wir die Bedeutung der Orange an ihre Form und Farbe knüpfen. Die Bedeutung der Orange aber liegt freilich zum Theil selbst mit in Form und Farbe, doch keineswegs allein, vielmehr in der Gesammtheit dessen, was sie ist und wirkt, insbesondere in Beziehung auf uns selbst ist und wirkt. Wennschon nun dem Sinn unmittelbar nur Form und Farbe präsent ist, so fügt die Erinnerung das Uebrige, nicht einzeln, aber in einem Gesammt-Eindrucke hinzu, trägt es in den sinn-

lichen Eindruck hinein, bereichert ihn damit, malt ihn so zu sagen damit aus; wir mögen das kurz die geistige Farbe nennen, die zur sinnlichen hinzutritt oder den associirten Eindruck, der sich mit dem direkten verbindet. Und darin liegt es, daß uns die Orange schöner als die gelbe Holzkugel erscheint.

In der That, sieht denn der, der eine Orange sieht, blos einen runden gelben Fleck in ihr? Mit dem sinnlichen Auge, ja; geistig aber sieht er ein Ding von reizendem Geruch, erquickendem Geschmack, an einem schönen Baume, in einem schönen Lande, unter einem warmen Himmel gewachsen in ihr; er sieht so zu sagen ganz Italien mit in ihr, das Land, wohin uns von jeher eine romantische Sehnsucht zog; aus der Erinnerung an all das setzt sich die geistige Farbe zusammen, womit die sinnliche verschönernd lasirt ist; indeß der, der eine gelbe Holzkugel sieht, eben blos trocknes Holz hinter dem runden gelben Flecke sieht, das in der Drechslerwerkstatt gedreht und vom Lackirer angestrichen ist. Beidesfalls associirt sich der aus der Erinnerung resultirende Eindruck so unmittelbar an die Anschauung, verschmilzt so vollständig damit, bestimmt so wesentlich den Charakter derselben mit, als wenn er ein Bestandtheil der Anschauung selbst wäre. Daher wir freilich leicht geneigt sein können, ihn mit als eine Sache derselben selbst zu rechnen, und nur durch Vergleiche, wie wir einen solchen eben anstellten, dahinter kommen können, daß er es nicht ist.

Ein anderes Beispiel:

Warum gefällt uns eine rothe Wange an einem jugendlichen Gesichte so viel besser als eine blasse? Ist es die Schönheit, der Reiz des Roth an sich? Unstreitig hat das Antheil daran. Ein frisches Roth erfreut das Auge mehr als Grau oder Mißfarbe. Man kann es sogar durch Erfahrung beweisen. Der Engländer Chesselden operirte einen Blindgebornen, der nie Farben gesehen hatte und bei dem sich also auch an Farben noch nichts associiren konnte. Dieser erklärte von vorn herein Scharlach für die schönste aller Farben; unter den übrigen gefielen ihm die muntersten am besten, wogegen Schwarz ihm großes Unbehagen erregte. Die Wilden, die ihren Körper bemalen, bemalen ihn am liebsten mit rother Farbe. Die frühesten Götterbilder aus Holz und Thon waren roth angemalt. Haben wir also nicht in dem natürlichen Reiz der rothen Farbe den einfachen Grund des Wohlgefallens an der rothen Wange? Aber, frage ich wieder, warum gefällt uns dann ein gleich frisches Roth an Nase und Hand nicht ebenso gut wie an der Wange? Es mißfällt uns vielmehr. Der wohlgefällige Eindruck des Roth muß also bei der Nase und Hand durch ein mißfälliges Element überboten werden. Worin kann das liegen? Es ist nicht schwer zu finden. Die rothe Wange bedeutet uns Gesundheit, Freude, blühendes Leben; die rothe Nase erinnert an Trunk und Kupferkrankheit, die rothe Hand an Waschen, Scheuern, Manschen; das sind Dinge, die wir nicht haben, noch treiben möchten. Wir möchten auch nicht daran erinnert sein.

Wäre umgekehrt von jeher die rothe Nase und blasse Wange als Zeichen der Gesundheit und Mäßigkeit, die blasse Nase und rothe Wange als Zeichen des Gegentheils erschienen, so würde auch die Richtung unseres Gefallens daran sich umkehren. Die Nordamerikanerinnen und Polinnen ziehen wirklich eine blasse Wange einer rothen vor, und suchen sich nöthigenfalls die blasse sogar auf Kosten ihrer Gesundheit durch Essigtrinken oder andere Mittel zu verschaffen. Meint man nun wohl, weil ihnen Blässe an sich besser gefällt als Röthe? Gewiß nicht, sondern weil sie sich gewöhnt haben, in der blassen Wange das Zeichen einer feinen Konstitution, höhern Bildung und Lebensstellung, in der rothen das einer blos bäuerlichen Gesundheit zu sehen, und ersteres letzterem vorziehen. Aus gleichem Grunde erscheinen den Chinesen verkrüppelte Füße an ihren Damen wohlgefällig,

die schönsten natürlichen bäuerlich plump, und geben sie ihren Götzen dicke Bäuche, weil sie gewohnt sind, die vornehmsten Würdenträger ihres Reiches mit dicken Bäuchen zu sehen, und die Vorstellung einer gewissen Erhabenheit über irdische Noth und Arbeit, welche es freilich zu dicken Bäuchen nicht kommen läßt, daran knüpfen.

Ich hörte einmal eine Dame sagen, man könne die Schönheit eines menschlichen Fußes doch eigentlich nur recht beurtheilen, wenn er beschuht sei. Gehörte nicht zu den Tugenden dieser Dame eine besondere Aufrichtigkeit, würde sie sich wahrscheinlich gescheut haben, diesen Ausspruch zu thun, so curios mag er den Meisten scheinen. Doch hat er etwas sehr Wahres. Wir lernen die Bedeutung des menschlichen Fußes fast nur kennen, während ihn der Schuh verbirgt; und sind nur über die Bedeutung des beschuhten Fußes recht orientirt. Nackt sehen wir ja fast nur den eigenen Fuß, der nicht immer der schönste ist, und den Fuß von Statuen, nach dem wir bei einer Statue am letzten zu sehen pflegen; also sind uns die Beziehungen des Fußes, die unser Gefallen daran mitbestimmen, beim nackten Fuße nicht ebenso geläufig wie beim beschuhten; und, während zur Beurtheilung der Schönheit des erstern eine gewisse Kunsterfahrung gehört, bedarf es zur Beurtheilung der Eleganz und Zierlichkeit des letztern nur der gewöhnlichen gesellschaftlichen Erfahrung.

Nicht minder als durch das Gebiet des Sichtbaren greift das Princip durch alle übrigen Sinnesgebiete durch.

Eine Blinde, welche sich der Formen nur durch den Tastsinn bemächtigen konnte, wurde gefragt, weßhalb ihr der Arm einer gewissen Person so wohl gefiele. Man rathet etwa: sie antwortete, weil sie den sanften Zug, die schöne Fülle, die elastische Schwellung der Formen des Armes fühle. Nichts von alledem; sondern weil sie fühle, daß der Arm gesund, rege und leicht sei. Das konnte sie aber nicht unmittelbar fühlen, sondern nur an das Gefühlte associiren. Nun glaube ich nicht, daß der direkte Eindruck, in dem man den alleinigen Grund ihres Wohlgefallens suchen mochte, wirklich ohne Antheil daran war; aber man sieht doch, daß der associative Eindruck ihr noch lebendiger zum Bewußtsein kam. Bei uns Sehenden ist es umgekehrt. Wir meinen, einem schönen Arme seine ganze Schönheit gleichsam abzusehen, ohne zu ahnen, daß wir das Meiste davon hinzusehen.

Eine Frau, die ihren Mann sehr liebte, sagte zu ihm: wie freue ich mich, daß du einen so hübschen Namen hast. Der Name war nicht sehr hübsch, aber sie liebte den Mann, darum gefiel ihr der Name. Ich selbst erinnere mich, daß mir als Kind der Name Kunigunde sehr wohlgefiel, bis ich ein Mädchen von fatalem Aussehen und Charakter mit diesem Namen kennen lernte, alsbald ward mir der Name fatal; und da mir seitdem keine besonders liebenswürdige Kunigunde begegnet ist, so ist der Eindruck geblieben.

Oekonomen pflegen den Geruch des Düngers wegen der Erinnerung an die Fruchtbarkeit, die er bringt, zu lieben.

Und was ließe sich nicht noch Alles in dieser Richtung anführen.

Aber, so höre ich mir von Oben herab zurufen: wozu dieser ganze Aufwand von Beispielen? was ist damit für die Aesthetik gewonnen, und überhaupt zu gewinnen? die Orange, die Wange, die Nase, die Hand, der Fuß u. s. w. sind unselbständige Theile der Natur und des Menschenkörpers; eine Aesthetik aber, die sich nicht niedrig halten will, geht vor Allem auf das Ganze, und zieht die Theile blos als solche in Betracht.

Wohl, so fassen wir die Bedeutung des Principes weiterhin auch für die Schönheit einer ganzen Landschaft, der ganzen Menschengestalt, eines ganzen Kunstwerkes in das Auge, und wir werden sie nicht geringer als für die Theile, sondern in demselben Verhältnisse erweitert und gesteigert wiederfinden, als das Ganze die Theile übersteigt. Es läßt sich nur das Princip am einfachsten an den einfachsten Beispielen erläutern, und wir

können auf unserem Wege von Unten nicht in der Richtung gehen, die für den Weg von Oben als der allein mögliche erscheint. Vorbehaltlich also, künftig höher aufzusteigen, fassen wir erst auf Grund der bisherigen Beispiele die Hauptgesichtspunkte des Princips wie folgt zusammen.

Jedes Ding, mit dem wir umgehen, ist für uns geistig charakterisirt durch eine Resultante von Erinnerungen an Alles, was wir je bezüglich dieses Dinges und selbst verwandter Dinge äußerlich und innerlich erfahren, gehört, gelesen, gedacht, gelernt haben. Diese Resultante von Erinnerungen knüpft sich ebenso unmittelbar an den Anblick des Dinges, wie die Vorstellung desselben an das Wort, womit es bezeichnet wird. Ja Form und Farbe des Dinges sind so zu sagen nichts als sichtbare Worte, welche uns die ganze Bedeutung des Dinges unwillkürlich vergegenwärtigen; wir müssen freilich diese sichtbare Sprache ebenso gut erst gelernt haben, um sie zu verstehen, wie die Sprache der Worte. Wir sehen einen Tisch, im Grunde nur einen viereckigen Fleck; aber in dem viereckigen Flecke Alles, wozu ein Tisch gebraucht wird; das macht den viereckigen Fleck erst zu einem Tische. Wir sehen ein Haus, aber in dem Hause alles mit, wozu ein Haus dient, was in einem Hause vorgeht; das macht erst den Fleck zu einem Hause. Wir sehen es nicht mit dem sinnlichen, aber mit einem geistigen Auge. Wir erinnern uns dabei nicht alles dessen einzeln, was zu dem Eindrucke beiträgt; wie wäre das möglich, wenn Alles zugleich Anspruch macht, in's Bewußtsein zu treten. Vielmehr, indem es das will, verschmilzt es zu dem einheitlichen gefühlsmäßigen Eindrucke, den wir die geistige Farbe nannten, ein Ausdruck, der in mehr als einer Hinsicht sehr bezeichnend ist. Mischen wir noch so viel verschiedenartige Farben zusammen, und das Gemisch macht doch immer wieder nur den einigen Eindruck einer Farbe, die sich aber nach den Farbebestandtheilen ändert, und, auf einen kompakten Farbengrund lasirend aufgetragen, abermals mit ihm einen einigen Eindruck giebt, der sich nach der Zusammensetzung von beiden richtet. So resultirt aus allen verschiedenartigen Erinnerungen, die sich an den Anblick eines Dinges knüpfen, doch immer nur ein einiger Eindruck, der aber nach der Zusammensetzung aus verschiedenen Erinnerungs-Ingredienzien verschieden ausfällt und mit dem direkten Eindruck des Anblicks auch wieder zu einem einigen Eindrucke verschmilzt. Nun kann selbst bei fast gleichem sinnlichen Eindrucke doch ein ganz verschiedener Totaleindruck durch die Ausmalung mit verschiedener geistiger Farbe entstehen, wobei ein kleiner sinnlicher Unterschied nur nöthig ist, die verschiedene Anknüpfung zu vermitteln. Eine Orange, gelbe Holzkugel, Messingkugel, Goldkugel, der Mond, alles für den Sinn nur runde, gelbe, nicht sehr verschieden aussehende Flecke, und doch wie verschieden der Eindruck, den sie machen! Vor der Goldkugel stehen wir mit einer Art kalifornischer Hochachtung, ganze Paläste, Kutsch' und Pferde, Bediente in Livree, schöne Reisen scheinen sich daraus zu entwickeln; die Holzkugel scheint nur zum Kollern da; und welch' hohe Idealität steckt in dem Monde! Nur dadurch auch gewinnt alles Aechte, der ächte Diamant, das ächte Gold, die ächten Spitzen, der ächte Nachtigallengesang, den ungeheuren Vortheil des Eindruckes vor allem noch so täuschend Nachgemachten.

Nach Maßgabe nun, als uns das gefällt oder mißfällt, woran wir uns bei einer Sache erinnern, trägt auch die Erinnerung ein Moment des Gefallens oder Mißfallens zum ästhetischen Eindrucke der Sache bei, was mit anderen Momenten der Erinnerung und dem direkten Eindrucke der Sache in Einstimmung oder Konflikt treten kann. Daraus gehen die mannigfachsten ästhetischen Verhältnisse hervor, die es interessant wäre, in Beispielen zu verfolgen; aber es würde für jetzt zu weit führen. Die stärksten und häufigsten Einwirkungen, die wir von einer Sache, in Verbindung mit einer Sache und vergleichs-

weise mit einer Sache erfahren, hinterlassen natürlich auch Erinnerungen, die am kräftigsten bestimmend in den associirten Eindruck eingreifen.

Erinnerungen sind einzeln genommen freilich immer verhältnißmäßig schwach gegen das, an was sie erinnern; aber indem viele Erinnerungen mit einem direkten Eindrucke zusammentreffen, kann ihre verschmolzene Wirkung die des direkten Eindruckes um so leichter überbieten, je inhaltreicher sie zugleich sind.

An was Alles erinnert nicht die Orange und wie interessant ist das, woran sie erinnert, gegen ihre bloße Form und Farbe!

Wie weit das Uebergewicht eines aus vergangenen Erfahrungen resultirenden, im Geiste gesammelten Eindruckes über den gegenwärtigen, den direkten Eindruck gehen kann, davon vermag uns ein alltägliches Beispiel zu belehren. Hält man einen Finger in doppelte Entfernung vor die Augen, so meint man, ihn noch genau ebenso groß zu sehen; und doch ist sein Bild in den Augen nur halb so groß, und kann er einem frisch operirten Blinden nur halb so groß erscheinen. Das aus unserer ganzen Lebenserfahrung fließende Wissen, daß er in jeder Entfernung gleich groß bleibt, übertäubt die sinnliche Erscheinung seiner Ungleichheit so ganz, daß wir ihn selbst mit den Augen in jeder Entfernung gleich zu sehen glauben. Ist dann zu verwundern, wenn wir auch die aus frühern Erfahrungen resultirende Wohlgefälligkeit vieler Dinge für Sache ihrer sinnlichen Erscheinung halten, die vielmehr Sache unserer geistigen Zuthat ist?

Habe ich bisher Gewicht darauf gelegt, daß im ästhetischen Totaleindrucke sich dessen verschiedene Elemente nicht scheiden, so muß doch die Aesthetik, um klare Rechenschaft von seinem Zustandekommen zu geben, solche scheiden, muß fragen: was ist Sache des direkten Eindruckes, was hängt an den Associationen, und was tragen diese oder jene dazu bei. Erschöpfend zwar kann eine solche Analyse niemals sein, weil im Allgemeinen unzählige Erinnerungen zu jedem associirten Eindrucke beitragen, ja streng genommen zu jedem der gesammte Erinnerungsnachklang unseres Lebens, nur mit einem anderen Gewichte seiner verschiedenen Momente. Schlagen wir einen Punkt eines gespannten Gewebes irgendwo an, — unser gesammter Vorstellungszusammenhang aber ist einem solchen Gewebe vergleichbar — so zittert das ganze Gewebe, nur die Punkte am stärksten, die dem angeschlagenen Punkte zunächst liegen und durch die stärksten Fäden damit zusammenhängen. Jede Anschauung aber schlägt sogar mehr als einen Punkt unseres geistigen Gewebes zugleich an. Doch kann man sich, unter Anerkennung dieses Zusammenwirkens unseres ganzen geistigen Besitzthums zu jedem Eindruck, die Aufgabe stellen, die Hauptmomente zu finden, die vorwiegend den Eindruck bestimmen. In unserer heutigen Aesthetik freilich findet man diese Aufgabe nicht erfüllt, weil nicht einmal den Gesichtspunkt derselben gestellt.

Um so mehr aber hat die Aesthetik Anlaß, auf die Komposition des Totaleindruckes aus seinen Elementen einzugehen, als einheitliche Eindrücke sich überhaupt nicht beschreiben, aber doch nach ihrer Zusammensetzung aus verschiedenen Komponenten charakterisiren lassen, wozu sich der Anlaß oft genug bietet. Wer will den Eindruck, den eine Orange, eine Goldkugel, eine Holzkugel macht, beschreiben? Dagegen läßt sich derselbe wohl durch die Vorstellungen, die sich dazu verschmolzen haben, charakterisiren.

Nicht blos aber durch die, die sich darin verschmolzen haben, sondern auch durch die, die wieder daraus hervortreten können, was einen neuen, wichtigen Gesichtspunkt darbietet. In der That können alle Vorstellungen, die zum geistigen Eindrucke beigetragen haben, auch unter Umständen wieder daraus hervortreten; es bedarf nur besonderer äußerer oder innerer Anlässe dazu. Das begründet die Möglichkeit, sich nach gewonnenem Totaleindruck eingehend nach verschiedenen, doch unter sich zusammenhängenden Richtungen mit dem Gegen-

stande zu beschäftigen, was einen zweiten递Haupttheil der ästhetischen Wirkung der Gegenstände bildet, die ja nicht blos in ihrem einheitlichen Totaleindrucke ruht. Dieser ist so zu sagen nur das Saamenkorn, aus dem eine ähnliche Pflanze sich zu entfalten vermag, als die, aus der es entstand. Zugleich ist jene Resultante von Erinnerungen der Quell, aus dem die Phantasie schöpft; und da neuerdings so häufig die ganze Schönheit durch Bezugnahme auf die Phantasie erklärt wird, so sollte hierin eine Aufforderung liegen, diesen Quell genauer zu untersuchen, als ich finde, daß es seither geschehen ist.

Indessen, so viel auf die geistige Farbe der Dinge zu geben, so ist doch nicht zu viel darauf zu geben, wozu man leicht verführt sein könnte, nachdem man einmal ihre Wichtigkeit erkannt hat. Denken wir uns an der Orange statt der schönen goldgelben eine graue unscheinbare Farbe, statt der reinen Rundung eine schiefe krüppeliche Form, so werden alle angeknüpften Erinnerungen sie nicht schön, nicht wohlgefällig erscheinen lassen. Eine Vanilleschote kann ähnliche Erinnerungen wecken, wie die Orange, und wer möchte sie schön aussehend finden! Aber deshalb darf man auch wieder nicht zu wenig auf den associirten Eindruck geben. Der Vergleich der Orange mit der Holzkugel verwehrt es. Weder der direkte noch der associirte Eindruck leisten viel für sich, aber sie leisten viel im Zusammenhange, geben zusammen ein höheres als blos additionelles Produkt des Wohlgefallens. Hier tritt wieder ein sehr allgemeines und weitgreifendes ästhetisches Princip, ich nenne es das der ästhetischen Steigerung, in Kraft, wovon man in der heutigen Aesthetik von Oben nichts erfährt. So wichtig es aber für die Beurtheilung der Weise ist, wie der direkte und associirte Eindruck im Totaleindrucke zusammenwirken, ist es doch für jetzt nicht möglich, in's Weitere darüber einzugehen.

Dies die allgemeinsten, schon an den einfachsten Beispielen erläuterbaren Gesichtspunkte des Principes, die nicht minder gültig bleiben, wenn wir uns nun zu Beispielen von höherer Stufe erheben.

Versuchen wir uns Rechenschaft von dem Eindrucke zu geben, den der Blick in eine Landschaft auf uns macht! Es ist etwas Unsagbares darin, etwas, was sich durch keine Beschreibung erschöpfen läßt. Wie wird man sich die Natur und die Gründe des Eindruckes erklären können? Um ein Beispiel der verschiedenen Weise zu geben, wie die Aesthetik von Oben und die Aesthetik von Unten überhaupt in ihren Erklärungen vorgehen, stelle ich eine Erklärung davon nach beiden einander gegenüber, die eine, im ersten Wege, geschöpft aus einem der geschätztesten neueren Lehrbücher der Aesthetik, dem von Carriere, die andere so, wie sie sich im zweiten Wege auf Grund unseres Principes ergiebt. Jenes die fernstliegende, an die höchsten idealsten Gesichtspunkte anknüpfende Erklärung, dieses die nächstliegende, an die untersten Gesichtspunkte anknüpfende Erklärung.

„Das Wesen der Natur — sagt Carriere (I. 243) — entspricht an sich der Schönheit; denn sie ist Erscheinung für den Geist, welchem sie in sinnfälligen Formen idealen Gehalt darstellt und geistige Gesetze veranschaulicht, und gerade das erfreut uns so innig, wenn in dem Aeußerlichen und Materiellen ein verwandtes Seelenvolles dem Gemüth entgegenkommt. Doch ist überall zunächst das eigene Leben des Lebens Zweck, jedes Wesen ist um seiner selbst willen da und nicht deswegen geschaffen, daß seine Gestalt uns ergötze; es ist eine Gunst des Schicksals, wenn in der Totalität des Universums das Wechselverhältniß der Dinge, die Art und Weise, wie sie für einander sind, uns für unseren Standpunkt gerade so sich darstellt, daß wir auf der sich uns bietenden Oberfläche doch das innere Wesen wahrnehmen und erkennen, wie die Formen der Dinge nicht blos den Zwecken des Alls entsprechen, sondern auch den Bedingungen und Forderungen unserer Persönlichkeit gemäß sind. Ja wir mögen ganz besonders die Güte und Herrlichkeit des Urgrundes

der Welt darin preisen, wenn Stoffe, die für das Leben des Organismus, namentlich der Pflanzen, gleichgültig erscheinen oder von ihm ausgeschieden werden, als ätherische Oele oder Pigmente durch Wohlgeruch oder Farbenglanz uns erquicken" u. s. w.

Und um auch zu zeigen, wie die Betrachtung des Einzelnen in diese allgemeine Betrachtung hineintritt, so wird (S. 258) von der Pflanze als Element der Landschaft gesagt:

„Die Potenzen der unorganischen Natur finden in der Pflanze einen Mittelpunkt des Zusammentreffens, indem hier eine individuelle Idee als leibgestaltende Lebenskraft auftritt und in der stets erneuten Bildung eines Organismus sich bethätigt, der durch die Wurzeln mit der Erde zusammenhängt, aber in Luft und Licht emporstrebt und mit Zweigen und Blättern nach der Seite sich ausbreitet. Die Pflanze veranschaulicht den Begriff des organischen Gestaltens, welchen wir früher für die Schönheit forderten, die Mannigfaltigkeit der Blätter und Zweige geht aus der Einheit hervor und wird sichtbar von ihr getragen, und die Wechselwirkung der einzelnen Gestalten schließt sich zu einem harmonischen Ganzen zusammen."

Gegen diesen Schwung der Betrachtung hat nun freilich unsere Betrachtung von Unten nichts Entsprechendes einzusetzen. Nehmen wir die folgende so simpel, wie sie sich giebt.

Dem Auge des Blindgeborenen, der nach glücklicher Operation das erste Mal in's Freie sieht, erscheint die ganze Natur nur erst als ein marmorirtes Blatt, denn er vermag noch nicht, in dem Gesehenen dessen Bedeutung mit zu sehen. Er sieht hinein in's Weite: da sind Wiesen, Felder, Wälder, Berge, Seen; er sieht nichts von Wiesen, Feldern, Wäldern, Bergen, Seen; er sieht nur grüne, gelbe, helle, dunkle Flecke. Nur das Gefühl des weittragenden Blickes, der sinnliche oder wenig über den sinnlichen hinaussteigende Reiz des Hellen und Dunklen, des Farbenkontrastes, der Mannigfaltigkeit, des Wechsels bestimmen den Eindruck, den er von der Landschaft hat. Aber ist das auch Alles, was w i r von der Landschaft haben? Wir haben das Alles auch, es trägt bei zu dem Eindrucke, den die Landschaft auf uns macht, der Stimmung, die sie uns erweckt, sogar nicht wenig dazu bei; aber wir sehen zugleich im fernen Walde, der für das unerfahrene Auge nur ein grüner Fleck ist, etwas, was lebendig in sich treibt und wächst, was Schatten, Kühlung giebt, worin der Hase, das Reh laufen, der Jäger geht, die Vögel singen, manch' Märchen spukt, auch wenn wir nichts wirklich davon sehen und hören. Im See, worin Jener nur einen blanken oder blauen Fleck erkennt, wissen wir, gehen die Wellen, spiegelt sich der Himmel, spielen die Fische, fahren die Schiffe u. s. w. Vorstellungen von Allem, was sonst treibt und wächst und wogt, klingen mit dabei an. Im Grunde sehen auch wir mit leiblichem Auge von Wald und See nicht mehr als der frisch operirte Blinde und das neugeborene Kind, das ist grüne und blanke oder blaue Flecke; Alles aber, was wir je von Wald und See gesehen, gehört, gelesen, erfahren, gedacht haben, wie Alles, womit sie einen Vergleichspunkt bieten, trägt zu dem Eindrucke bei, den diese Gegenstände auf uns machen, und macht ihren Anblick dadurch zu etwas unsäglich Bedeutenderem, Reicherem, Lebendigerem, für das Gefühl Vertiefterem, für die Phantasie Produktiverem, als für den, der nichts davon gesehen, gehört, gedacht hat. Und wie es mit Wald und See ist, ist es mit allen Elementen der Landschaft, Wiese, Feld, Berg, Haus. An Alles knüpfen sich Erinnerungen, Vergleichs-Vorstellungen, wodurch diese Gegenstände eine gewisse Bedeutung für uns erlangen, und auch ihre Zusammenstellung gewinnt für uns eine solche auf demselben Wege. Die Gesammtheit dieser Erinnerungen und Vorstellungen nun macht sich in Verschmelzung mit der sinnlichen Unterlage und den ihr

immanenten Verhältnissen als Gesammteindruck der Landschaft geltend; jede Einzelnheit der Landschaft spielt von einer anderen Seite mit einem anderen Kreise von Erinnerungen und Vorstellungen hinein, und was so hineinspielt, kann auch wieder herausspielen.

Hiernach versteht sich leicht, worin das Unsagbare, Unerschöpfliche, Unklärbare liegt, was dem landschaftlichen Eindrucke zukommt. Wer will alle Vorstellungen verfolgen, erschöpfen, klären, die dazu beigetragen haben? Schon dem einzelnen Gegenstande kommt eine gewisse Unerschöpflichkeit in dieser Hinsicht zu; die Landschaft bietet uns so zu sagen eine Unerschöpflichkeit solcher unerschöpflicher Gegenstände dar, die mit ihren Associationskreisen sich ineinander unbestimmt verzweigen. Doch können wir auch hier Hauptelemente in Betracht ziehen und den Eindruck dadurch wenigstens bis zu gewissen Grenzen charakterisiren, klären und erklären. Zum Beispiel:

Es wird wohl Jedem aufgefallen sein, welchen Reiz, welche Bedeutung eine sonst unbedeutende Gegend oft durch ein am rechten Flecke stehendes Haus, ein Schloß, ein Dörfchen erhalten kann; man denke z. B. an das Schloß in Wernigerode, das Dorf Gernrode am Fuße des Stubenberges, die Häuser unten in Wilhelmsthal. Das Menschenwerk aus den Landschaften dieser Oertlichkeiten wegdenken, heißt ihnen die Pointe nehmen. Nun kann man wohl etwas von dem Reize dieser Komposition aus Architektur und Natur in der Verbindung, dem Wechsel und Gegensatz ihrer verschiedenartigen Formen und Farben finden; aber wie wenig würde das ohne Rücksicht auf die angeknüpfte Bedeutung dieser Verbindung, dieses Wechsels, dieses Gegensatzes sagen! Die Hauptsache liegt darin, daß die Erinnerung an das Menschliche, die Beziehung des Menschen zur Natur, die sich theils als eine Herrschaft über die Natur, theils als ein Gegensatz gegen die Natur, theils als ein Verkehr und Leben mit und in der Natur geltend macht, bereichernd zum direkten Eindruck hinzutritt.

Nun darf man nicht sagen, obwohl man es zu mir gesagt hat: alles das ließe sich auch ohne die Anschauung des Bauwerkes in der Natur durch bloße Vorführung in der Vorstellung haben; doch würde man damit den landschaftlichen Eindruck des Bauwerkes in der Natur nicht haben; also kann er nicht auf solchen Associationen ruhen. — Aber, was man sich einzeln, nach einander, unvollständig, mit der Mühe der Ueberlegung, ohne wesentlich verknüpfendes Band vorführen möchte, wird uns mit einem Schlage in einem Gesammteindrucke durch die Anschauung des Bauwerkes in der Natur, als wie ein Bestandtheil dieser Anschauung selbst, geschenkt. Das ist doch etwas sehr Anderes, als jene Vorführung, und daran kann auch ein sehr anderer Eindruck hängen.

Ich will hierzu eines kleinen Beispiels eigener Erfahrung gedenken, wo mir das Alles recht lebhaft entgegentrat.

In den vorigen Ferien brachte ich mit meiner Frau einige Wochen in einem Försterhause, eine Viertelstunde von Lauterberg im Harze, zu. Unserer Wohnung gegenüber war ein grüner Abhang, den wir oft erstiegen, und von wo wir die Aussicht über eine weite waldige Berglandschaft von wenig entwickelten Formen hatten. Außer dem Försterhause im Vordergrunde waren nirgends menschliche Wohnungen zu sehen; nur in der Ferne ragte aus der Monotonie des an den Bergen lehnansteigenden grünen Waldes ein einziges rothes Dach hervor. Dieses aber brachte einen ganz eigenen Reiz in die sonst einfachen Stimmungsverhältnisse der Aussicht. Es war eben die Pointe der ganzen Landschaft. Und ich sagte mir: wie, wenn man ein ganz eben solches rothes Fleckchen auf eine grüne Wand machte, würde es auch eben so idyllisch, sentimental, romantisch, märchenhaft aussehen, wie das rothe Dach in der Wald-Landschaft? Gewiß nicht. Aber könnte mir das rothe Fleckchen auf der grünen Wand auch wohl ebenso das Leben und Weben des Menschen mit

seinen Leiden und Freuden in einer einsamen Waldnatur auf einmal vergegenwärtigen, wie das rothe Dach im Walde?

Doch wieder eines Einwurfes habe ich zu gedenken, den mir Jemand bei Gelegenheit dieses Beispiels machte, Jemand, der, in der Schule der neueren Aesthetik erzogen, die Einführung einer neuen Gottheit in sie, wofür er das Associationsprincip hielt, nicht dulden wollte.

All' das, sagte er, was die Erinnerung zum Eindrucke des rothen Daches und grünen Waldes hinzubrachte, was sich von Nebenvorstellungen anknüpfte, gehört gar nicht zum Wesen des ästhetischen, des wahrhaft landschaftlichen Eindruckes und wäre erst abzusondern, um ihn rein zu haben. Denn der reine landschaftliche Eindruck, um dessen Hervorrufung es insbesondere dem Künstler zu thun ist, ruht doch nur in den eigenen so zu sagen musikalischen Verhältnissen der Form und Farbe, die durch das Auge direkt in uns eingehen, und womit wir das wirklich Sichtbare, wie das Dach zum Hause, die grüne Waldfläche zum Walde in der Vorstellung ergänzen. Nur was Haus und Wald nach ihrem eigenen sichtbaren Wesen sind und wie sie damit in die übrigen Verhältnisse der Sichtbarkeit eingreifen, kommt für ihren landschaftlichen Eindruck in Betracht.

Aber diesem Einwurfe liegt die Täuschung zu Grunde, daß Haus und Wald ihrem ganzen eigenen sichtbaren Wesen nach erheblich mehr als bedeutungslose und bedeutungslos in die Verhältnisse der Sichtbarkeit eingreifende, mit Farben ausgefüllte, Lineamente sind. Erst die Brauchbarkeit des Hauses zum Wohnen, erst das Vermögen des Baumes zum Wachsen, und was an beidem hängt, bringt Inhalt, Leben, Tiefe in den Eindruck dessen, was wir davon sehen; und das davon absondern, ist so gut, als ob man vom Menschen das lebendige Fleisch absonderte, um seine wesentliche Bedeutung im bloßen Skelett zu suchen. Wie mag der Einwurf mit dem landschaftlichen Eindruck einer Ruine zurecht kommen? Sollte dieser vielmehr von dem Gegensatze ihrer grauen Formlosigkeit gegen die Farben und Linien der Umgebung abhängen, als davon, daß die Ruine wie ein Brennpunkt von Erinnerungen in der lebendigen Gegenwart wirkt, so bedürfte es für den grauen zerklüfteten Felsen nicht erst der darauf stehenden Ruine, um denselben Eindruck zu machen. Und wie kann von einem romantischen, idyllischen, historischen Charakter der Landschaft überhaupt noch die Rede sein, wenn nicht das, was die Verhältnisse der Sichtbarkeit für das ganze Leben des Menschen bedeuten, ihnen erst die höhere malerische Bedeutung über den immerhin anzuerkennenden gegensätzlichen, harmonischen und rhythmischen Verhältnissen der Farben und Formen verliehe. So weit diese in Betracht kommen, gewinnen sie selbst erst durch Aufnahme in jene höheren Beziehungen höhere landschaftliche Bedeutung, und sind dann freilich nach dem Steigerungsprincipe als Träger des Höheren auch mit höherem Werthe als für sich zu veranschlagen. Doch, streiten wir uns lieber ein anderes mal darüber weiter, um noch an einige andere Erfahrungen zu erinnern.

Viele Schlösser und Klöster auf Hügeln und Bergen sind jetzt zu Irrenhäusern und Zuchthäusern eingerichtet; so wie wir dies erfahren, ist es, als ob der Reiz, den sie der Landschaft verliehen, mit kaltem Wasser ausgelöscht würde.

Man darf wohl sagen, daß jetzt zu den großartigsten Leistungen in der Baukunst die Eisenbahngebäude gehören. Welch' grandiose, charakteristische, in den reinsten Formen architektonischen Ebenmaßes gehaltene Werke dieser Art sieht man nicht nur an einem, sondern an vielen Orten! Dazu können sie die vollendetste Zweckmäßigkeit zeigen, und wer kennt nicht die große Rolle, welche die Zweckmäßigkeit, im Grunde auch nur durch Association, in der Aesthetik der Baukunst spielt? Doch mangelt dem Eindrucke dieser Gebäude immer etwas an der vollen Befriedigung und letzten Höhe; doch gewähren sie nie den erfreuenden Ein

druck eines Palastes oder den erhabenen eines Tempels. Warum? weil wir in ihnen den Schauplatz eines Trubels und geschäftsmäßigen Treibens sehen, das uns mißfällt.

Gehen wir über die Landschaft und die Architektur noch höher hinauf! Als das schönste Werk der Schöpfung gilt uns die menschliche Gestalt; die höchsten Werke der bildenden Kunst haben sie zum Gegenstande oder Elemente. Nun liegt unstreitig in dem Flusse der Formen, der zweiseitigen Symmetrie, vielleicht den einfachen Proportionen, wie manche wollen, oder gewissen rhythmischen Verhältnissen, wie Andre wollen, oder dem goldnen Schnitt, wie Zeising will, auch wohl in etwas Instinktivem Viel, was uns schon bei der einzelnen Gestalt, abgesehen von aller angeknüpften Bedeutung, gefallen kann; wozu beim ganzen Gemälde noch die Verhältnisse der Gruppirung und Farbengebung treten, in denen sich wohl auch etwas von harmonischen und disharmonischen Beziehungen an sich geltend machen kann. Aber alles das ist doch nur die niedere Unterlage für den sich anknüpfenden Ausdruck der Tauglichkeit der Menschengestalt zu den Geschäften und Freuden des Lebens und den noch höheren Ausdruck der Seele und der Seelenbewegungen, was Alles wir schon in der einzelnen Gestalt finden können, endlich für die allgemeineren und höheren menschlichen, ja über das Menschliche hinausreichenden Beziehungen, die wir im ganzen Gemälde finden können. Alles das aber tragen wir erst in die gesehenen Formen- und Farbenzusammenstellungen hinein, nach Erfahrungen über die Bedeutung derselben, die wir gemacht haben; alles das ist Sache des associirten, nicht des direkten Eindruckes. Nun soll man jene niedere Unterlage der Menschenschönheit so wenig verachten, als Versmaß, Rhythmus, Reim in dem Gedichte; aber auch nicht höher und in keinem andern Sinne achten; und wer kann die höchste Schönheit eines Gedichtes in Versmaß, Rhythmus, Reim suchen, wenn schon eine Verletzung davon die ganze Schönheit des Gedichtes eben so schänden, wie ihr reiner Fluß sie hoch heben kann? Wir haben hier eben wieder ein Beispiel der Wirkung des ästhetischen Steigerungsprincips, wonach das Niedere und das darauf gebaute Höhere ein größeres und höheres Produkt des Wohlgefallens geben können, als der Summe des Wohlgefallens am Niederen und Höheren für sich entspricht. Nicht anders aber mit der Schönheit des Menschen, als des Gedichtes.

Der Beitrag, den die Zweckmäßigkeit eines Bauwerkes zur Schönheit des Bauwerkes, der geistige Ausdruck zur Schönheit eines Menschen zu geben vermag, ist in jeder Aesthetik anerkannt; und damit indirekt auch die Leistung des Associationsprincipes anerkannt, nur damit nicht auch das Princip als Grund dieser Leistung anerkannt, noch die wesentlichen Gesichtspunkte dieses Principes damit erkannt. Oft wird der geistige Ausdruck als ein den Formen fertig anhängender oder von selbst sich daraus ergebender gefaßt, oft zum direkten Eindrucke geschlagen, was Sache der Association, oft, wie wir vorhin ein Beispiel hatten, deren wesentlicher Antheil am höheren ästhetischen Eindrucke ganz verkannt, ja geleugnet, oft auch untriftig die Schönheit allein in Zweck und Bedeutung gelegt. Viel ließe sich über alles dieß sagen, doch haben wir uns im Ganzen beschränkt, die Wege nicht zu gehen, die wir nicht für die besten halten, und schließen mit einigen allgemeinen Betrachtungen, wie wir begonnen.

Von vorn herein versteht sich, daß, wenn das Wohlgefallen an den Dingen wesentlich mit auf der Erinnerung an Wohlgefälliges beruht, es auch an sich Wohlgefälliges geben muß; und die Hauptaufgabe der Aesthetik von Unten beruht darin, die an sich bestehenden Quellen der Lust, des Wohlgefallens, wie die Gesetze ihres Zusammenwirkens festzustellen, und wo möglich zu einer allgemeinsten Quelle, einem allgemeinsten Gesetze, wie Lust und Unlust entstehen, aufzusteigen, bis jetzt freilich ein ebenso ungelöstes Problem, wie das Problem eines allgemeinsten Kraftgesetzes in der Physik. Auch haben wir uns hier keine

so allgemeine Aufgabe gestellt, sondern eben blos die, zu zeigen, daß unter den verschiedenen Quellen des Wohlgefallens die immerhin sekundär zu nennende der Association eine der wichtigsten Rollen spielt, dadurch spielt, daß sie Zuflüsse aus allen Quellen, die ursprünglicher als sie sind, in sich aufnimmt und in sich verschmilzt.

Man hat die direkten Quellen der Lust, des Wohlgefallens nicht schlechthin mit sinnlichen zu verwechseln, obwohl alle rein sinnlichen zu den direkten gehören, und hat die höheren ästhetischen Eindrücke nicht allein im Gebiete der Association zu suchen. Die Auffassung der Symmetrie, der Farbenverhältnisse, der Tonverhältnisse übersteigt das rein sinnliche Gebiet, ohne deßhalb eine Sache der Association zu sein. Alle höheren ästhetischen Eindrücke hängen überhaupt an Zusammenhängen und Beziehungen, die nun aber eben sowohl innere als äußere des Gegenstandes sein können. Hier spielen die inneren, dort die äußeren oder associativen die Hauptrolle.

Im Reiche des Sichtbaren kommt überhaupt kein ästhetischer Eindruck von einiger Höhe ohne das Associationsprincip zu Stande. Das Höchste, wozu es dieses Reich abgesehen davon bringt, ist die kaleidoskopische Figur und das Feuerwerk. Auch die Poesie gipfelt im associativen Faktor, denn der Sinn des Gedichtes ist nur angeknüpft an die Worte, und Versmaß, Rhythmus, Reim gewinnen erhebliche Bedeutung nur nach Maßgabe, als sie hierin eingehen. Hingegen spielt die Association in der Musik blos die Nebenrolle. Der höhere Eindruck der Musik beruht wesentlich auf einem Verfolgen ihrer inneren Beziehungen, und so viel sich daran anknüpfen kann, es ist dem eigentlichen musikalischen Eindrucke zufällig. Im Streben, einheitliche Principien aufzustellen, hat man mehrfach den Haupteindruck des Gemäldes in demselben Sinne von seinen inneren Beziehungen abhängig gemacht, wie den der Musik; aber die Malerei ist in dieser Beziehung verwandter mit der Poesie als der Musik, wenn schon nicht in jeder Hinsicht damit vergleichbar. Es würde von Interesse sein, diese theils analoge theils verschiedene Rolle des Associationsprincipes in den verschiedenen Künsten eingehender zu betrachten; doch immer von Neuem haben wir uns der Beschränkung zu erinnern, die uns hier auferlegt ist.

Meint man, die Schönheit werde dadurch, daß sie großentheils auf Associationen ruht, nach deren Zufälligkeit selbst eine Sache der Zufälligkeit, so ist das aus doppeltem Gesichtspunkte nicht triftig. Einmal, sofern sich nach den angeborenen und auf deren Grund entwickelten Bezugsverhältnissen der Menschen und Dinge ein gewisser Kreis von Associationen um jedes Ding n o t h w e n d i g entwickelt; zweitens, weil unter denen, die sich nach zeitlichen und örtlichen Umständen verschieden gestalten, doch nur eine gewisse Gestaltung die günstigst mögliche für die Menschheit ist, und daran hat sich der Begriff der wahren Schönheit zu halten. Das freilich folgt nothwendig aus dem Principe und wird sich dem Begriffe einer absoluten Schönheit gegenüber, den Manche haben, unschwer vertreten lassen, daß die Schönheit der menschlichen Gestalt nicht überall die höchste sichtbare Schönheit sein kann. Giebt es anders auf andern Weltkörpern Geschöpfe, die, den andern kosmischen Verhältnissen angemessen, anders als die Menschen organisirt und gebaut sind, so wird dort als höchste Schönheit die der Geschöpfe gelten, an die sich dort die werthvollste Bedeutung knüpft.

Dadurch, daß die Schönheit für den Menschen wesentlich mit auf Associationen dessen, was für ihn höhere und werthvolle Bedeutung hat, an die sinnliche Unterlage seines Lebens beruht, greift sie mächtig in die gesammten höheren Kulturverhältnisse der Menschheit ein, wie sie in gewissem Sinne erst daraus geboren wird, und gewinnt damit eine, über das unmittelbare Gefallen an ihr weit und hoch hinausreichende Bedeutung.
